# Speciation of Transition-Metal-Substituted Keggin-Type Silicotungstates Affected by the Co-crystallization Conditions with Proteinase K

**DOI:** 10.1021/acs.inorgchem.1c02005

**Published:** 2021-09-16

**Authors:** Joscha Breibeck, Elias Tanuhadi, Nadiia I. Gumerova, Gerald Giester, Alexander Prado-Roller, Annette Rompel

**Affiliations:** ‡ Universität Wien, Fakultät für Chemie, Institut für Biophysikalische Chemie, Althanstraße 14, 1090 Wien, Austria; § Universität Wien, Fakultät für Geowissenschaften, Geographie und Astronomie, Institut für Mineralogie und Kristallographie, Althanstraße 14, 1090 Wien, Austria; ∥ Universität Wien, Fakultät für Chemie, Institut für Anorganische Chemie und Zentrum für Röntgenstrukturanalyse, Währinger Straße 42, 1090 Wien, Austria

## Abstract

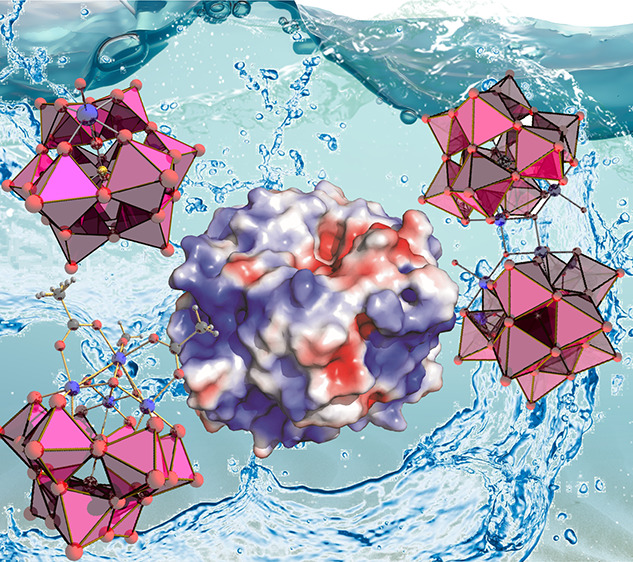

We report on the synthesis of the tetrasubstituted sandwich-type Keggin silicotungstates as the pure Na salts Na_14_[(A-α-SiW_10_O_37_)_2_{Co_4_(OH)_2_(H_2_O)_2_}]·37H_2_O (**Na{SiW**_**10**_**Co**_**2**_**}**_**2**_) and Na_14_[(A-α-SiW_10_O_37_)_2_{Ni_4_(OH)_2_(H_2_O)_2_}]·77.5H_2_O (**Na{SiW**_**10**_**Ni**_**2**_**}**_**2**_), which were prepared by applying a new synthesis protocol and characterized thoroughly in the solid state by single-crystal and powder X-ray diffraction, IR spectroscopy, thermogravimetric analysis, and elemental analysis. Proteinase K was applied as a model protein and the polyoxotungstate (POT)–protein interactions of **Na{SiW**_**10**_**Co**_**2**_**}**_**2**_ and **Na{SiW**_**10**_**Ni**_**2**_**}**_**2**_ were studied side by side with the literature-known K_5_Na_3_[A-α-SiW_9_O_34_(OH)_3_{Co_4_(OAc)_3_}]·28.5H_2_O (**{SiW**_**9**_**Co**_**4**_**}**) featuring the same number of transition metals. Testing the solution behavior of applied POTs under the crystallization conditions (sodium acetate buffer, pH 5.5) by time-dependent UV/vis spectroscopy and electrospray ionization mass spectrometry speciation studies revealed an initial dissociation of the sandwich POTs to the disubstituted Keggin anions H_*x*_Na_5–*x*_[SiW_10_Co_2_O_38_]^3–^ and H_*x*_Na_5–*x*_[SiW_10_Ni_2_O_38_]^3–^ (**{SiW**_**10**_**M**_**2**_**}**, M = Co^II^ and Ni^II^) followed by partial rearrangement to the monosubstituted compounds (**α-{SiW**_**11**_**Co}** and **α-{SiW**_**11**_**Ni}**) after 1 week of aging. The protein crystal structure analysis revealed monosubstituted α-Keggin POTs in two conserved binding positions for all three investigated compounds, with one of these positions featuring a covalent attachment of the POT anion to an aspartate carboxylate. Despite the presence of both mono- and disubstituted anions in a crystallization mixture, proteinase K selectively binds to monosubstituted anions because of their preferred charge density for POT–protein interaction.

## Abstract 2

We report on the development of a new synthesis protocol to prepare the Na salts of the tetrasubstituted sandwich-type Keggin derivatives Na_14_[(A-α-SiW_10_O_38_)_2_{Co_4_(OH)_2_(H_2_O)_2_}]·37H_2_O (**Na{SiW**_**10**_**Co**_**2**_**}**_**2**_) and Na_14_[(A-α-SiW_10_O_38_)_2_{Ni_4_(OH)_2_(H_2_O)_2_}]·77.5H_2_O (**Na{SiW**_**10**_**Ni**_**2**_**}**_**2**_). Following a thorough characterization of the polyoxotungstate (POT) dimers involving single-crystal and powder X-ray diffraction, IR spectroscopy, thermogravimetric analysis, and elemental analysis in the solid state and UV/vis spectroscopy and electrospray ionization mass spectrometry in solution, the water-soluble compounds (>5 mM) were applied as additives for the crystallization of proteinase K along with the tetrasubstituted monomeric Keggin-type analogue K_5_Na_3_[A-α-SiW_9_O_34_(OH)_3_{Co_4_(OAc)_3_}]·28.5H_2_O (**{SiW**_**9**_**Co**_**4**_**}**). Crystallographic studies on the obtained protein crystals revealed monosubstituted Keggin derivatives in all three cases bound to conserved sites of the protein, which highlights a selectivity of proteinase K toward monosubstituted Keggin POTs within a narrow range of surface charge density.

Polyoxometalates (POMs) are molecular oxo anions formed by early transition metals such as V, Mo, and W in high oxidation states.^1,2^ POMs, particularly polyoxotungstates (POTs), have been successfully applied in protein crystallography^3^ as stabilizing additives with a strong anomalous signal contribution to solve the phase problem.^4^ Previous studies on the Anderson-type POT [TeW_6_O_24_]^6–^ (TEW) revealed an increase of the entropic gain by releasing surface-bound hydration water^5^ and by mediating new crystal contacts^6^ to be the driving force for the co-crystallization process with proteins. Even novel crystal types^7−11^ and unprecedented POT structures can be obtained when TEW^12−15^ is applied as a crystallization additive. Following these pioneering studies, our group explored the potential of the Keggin archetype as a crystallization additive (Figure S1). A series of monosubstituted α-Keggin POTs, [α-PW_11_O_39_{TM(H_2_O)}]^5–^ (TM = Co^II^, Ni^II^, Cu^II^, and Zn^II^), was recently successfully applied for co-crystallization with proteinase K,^16^ involving the covalent interaction of a Co^II^ or Ni^II^ center with an aspartate side chain.^17^ This approach mimicked the bioaffinity separation principle of immobilized metal chelate affinity chromatography^18^ with a special focus on the identification of POT binding positions for the applied Keggin-type derivatives on the protein surface. To further develop the immobilization approach in the present study, the degree of metal substitution in Keggin POTs was raised, with the potential for multiple attachment or cross-links to the protein through different transition-metal sites within the same POT anion. Proteinase K (from *Tritirachium album*) served as an established model protein with a high ratio of basic residues (p*I* ≈ 8.9;^19^Figures S15 and S16).

Herein, we report the synthesis and characterization of two tetrasubstituted sandwich-type Keggin derivative POT structures, Na_14_[(A-α-SiW_10_O_37_)_2_{Co_4_(OH)_2_(H_2_O)_2_}]·37H_2_O (**Na{SiW**_**10**_**Co**_**2**_**}**_**2**_; Figure S5A) and Na_14_[(A-α- SiW_10_O_37_)_2_{Ni_4_(OH)_2_(H_2_O)_2_}]·77.5H_2_O (**Na{SiW**_**10**_**Ni**_**2**_**}**_**2**_; Figure S5B), whose solution stability was carefully investigated prior to crystallization studies. Given the complexity of biological media and the possible influence of buffer components on POMs,^20^ a detailed understanding of POM speciation under experimental conditions is of paramount importance. Co-crystallization was carried out side by side with the literature-known tetrasubstituted K_5_Na_3_[A-α-SiW_9_O_34_(OH)_3_{Co_4_(OAc)_3_}]·28.5H_2_O^21^ (**{SiW**_**9**_**Co**_**4**_**}**; Figure S5C), exhibiting acetate groups that suggest replacement by carboxylate side chains or other ligands on the protein surface.

Recently, Cs salts of tetrasubstituted sandwich Keggin silicotungstates have been reported.^22^ However, different routes for the synthesis of **{SiW**_**10**_**M**_**2**_**}**_**2**_ have been used. While the structures reported by Haider et al. were synthesized at room temperature with a starting ratio of 1:1 for the precursor Na_10_[A-α-SiW_9_O_34_]^23^ (**{SiW**_**9**_**}**) to a transition metal, our synthesis starts from a stoichiometric ratio of **{SiW**_**9**_**}**:M^II^ = 1:3 followed by subsequent heating to 80 °C for 1 h (Figure 1A). Considering that the high water solubility of POT salts is a prerequisite to studying their interactions with proteins in solution and as co-crystallization agents, our synthesis protocol includes an additional cation exchange in water, leading to the pure Na salts of **Na{SiW**_**10**_**Co**_**2**_**}**_**2**_ and **Na{SiW**_**10**_**Ni**_**2**_**}**_**2**_ with increased water solubility of more than 5 mM, relatively high for this POT class.

**Figure 1:**
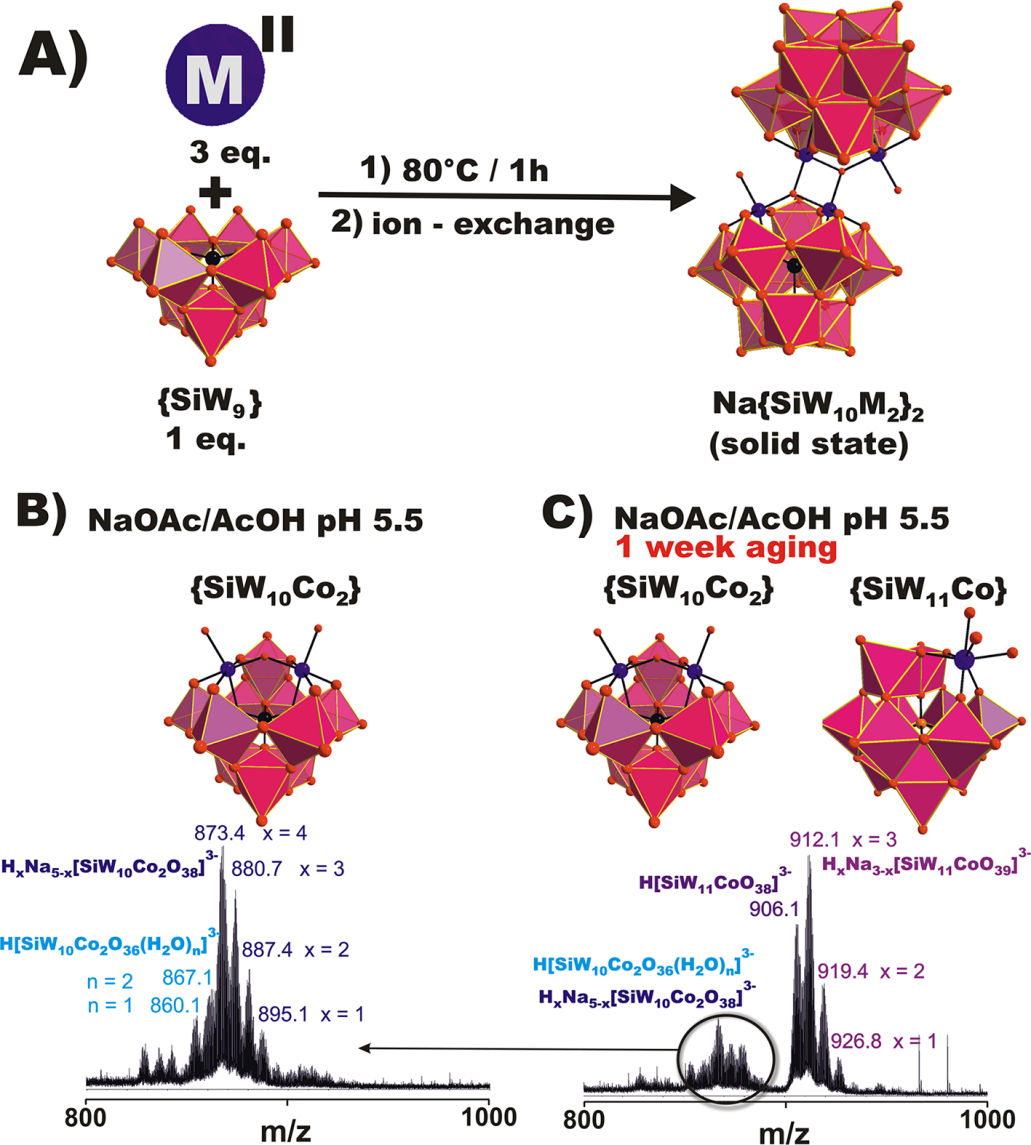
(A) Schematic representation showing the synthesis of **Na{SiW**_**10**_**M**_**2**_**}**_**2**_. Heating of the reaction mixture to 80 °C for 1 h and subsequent removal of excess transition metal M^II^ via ion exchange gives the product. (B and C) Results of ESI-MS studies exemplified on **Na{SiW**_**10**_**Co**_**2**_**}**_**2**_. The species are, in solid state, dimer **Na{SiW**_**10**_**Co**_**2**_**}**_**2**_, in the acetate buffer on the day of preparation, disubstituted monomer **{SiW**_**10**_**Co**_**2**_**}**, in a solution aged for 1 week, a mixture of disubstituted **{SiW**_**10**_**Co**_**2**_**}** and monosubstituted **{SiW**_**11**_**Co}**. The mass spectra for **Na{SiW**_**10**_**M**_**2**_**}**_**2**_ in the region *m*/*z* 200–1800 recorded in the negative mode are shown in Figures S13 and S14. Black, blue, and red spheres represent Si^IV^, M^II^, and O, respectively. Magenta octahedra are {WO_6_}.

Single-crystal X-ray diffraction (SXRD) studies (CCDC 2039844 and 2039852; Table S4) revealed that **Na{SiW**_**10**_**Ni**_**2**_**}**_**2**_ and **Na{SiW**_**10**_**Co**_**2**_**}**_**2**_ crystallize in the triclinic space group *P*1̅, whereas the Cs salts reported by Haider et al. crystallize in monoclinic crystal systems. **Na{SiW**_**10**_**Ni**_**2**_**}**_**2**_ and **Na{SiW**_**10**_**Co**_**2**_**}**_**2**_ were characterized by powder X-ray diffraction (PXRD; Figures S6 and S7) and IR spectroscopy (Figure S2 and Table S1) showing the terminal W=O and bridging W–O–W vibrations typical for the Keggin-type POT. The number of water molecules in **Na{SiW**_**10**_**Co**_**2**_**}**_**2**_·37H_2_O (Figure S3 and Table S2) and **Na{SiW**_**10**_**Co**_**2**_**}**_**2**_·77.5H_2_O was determined using thermogravimetric analysis (TGA; Figure S4 and Table S3).

To study the compounds’ behavior in solution, UV/vis spectroscopy and electrospray ionization mass spectrometry (ESI-MS) were applied. The application of more informative ^183^W or ^29^Si NMR spectroscopic studies is hampered by the high amount of paramagnetic transition-metal ions interfering with the signal intensity and resolution. The UV/vis spectra of **Na{SiW**_**10**_**Ni**_**2**_**}**_**2**_ and **Na{SiW**_**10**_**Co**_**2**_**}**_**2**_ show an absorption maximum at ∼221 nm, with a shoulder at ∼250 nm attributed to the pπ(O_b_) → dπ*(W) ligand-to-metal charge-transfer (LMCT) transitions typical for the Keggin POTs (Figure S8A).^22,24^ The visible spectrum of **Na{SiW**_**10**_**Co**_**2**_**}**_**2**_ displays a peak located at ∼512 nm, which is typical for octahedrally coordinated Co^II^ centers (Figure S8B).^22,25,26^ Considering the pronounced peak at ∼512 nm, time-dependent UV/vis studies were performed in water at pH 6.8 on **Na{SiW**_**10**_**Co**_**2**_**}**_**2**_ in the presence and absence of proteinase K, thereby circumventing potential peak overlap with the protein (Figure S12). A negligible decrease in absorption can be observed over 240 min for the POT in water in the absence of protein (Figure S11A), whereas a dramatic drop in absorption at ∼512 nm in the presence of proteinase K indicates strong POT–protein interactions via the Co^II^ site upon the formation of POT–protein adducts, which eventually leads to precipitation (Figure S11B). Moreover, time-dependent UV/vis studies on solutions of **Na{SiW**_**10**_**Co**_**2**_**}**_**2**_ in 100 mM NaOAc/AcOH (pH 5.5) show a pronounced decrease of the shoulder at ∼250 nm after incubation for 120 min (Figure S10A), pointing toward POT rearrangement. The UV/vis spectrum of **{SiW**_**9**_**Co**_**4**_**}** in NaOAc/AcOH (pH 5.5) is shown in Figure S9, demonstrating characteristic absorption in the UV/vis and near-IR regions attributed to pπ(O_b_) → dπ*(W) LMCT and d–d transitions for Co^II^. Time-dependent UV/vis studies on solutions of **{SiW**_**9**_**Co**_**4**_**}** in 100 mM NaOAc/AcOH (pH 5.5) show a decrease in the maximum at ∼196 nm and the appearance of a shoulder at ∼230 nm after incubation for 20 h (Figure S10B), which indicates a rearrangement of **{SiW**_**9**_**Co**_**4**_**}**. To further clarify the POT species present in solution, ESI-MS spectra of **Na{SiW**_**10**_**Ni**_**2**_**}**_**2**_ and **Na{SiW**_**10**_**Co**_**2**_**}**_**2**_ in water and acetate buffer (pH 5.5) were recorded in negative mode at the day of preparation, showing only signals of disubstituted monomeric anions H_*x*_Na_5–*x*_[SiW_10_M_2_O_38_]^3–^ (**{SiW**_**10**_**M**_**2**_**}**, M = Co^II^ and Ni^II^, *x* = 1–4; Figures S13 and S14), proving dissociation of the sandwich compounds (Figure 1B). The speciation remained unchanged in water for **Na{SiW**_**10**_**Ni**_**2**_**}**_**2**_ and **Na{SiW**_**10**_**Co**_**2**_**}**_**2**_ after 1 week (Figure S13B and S14B), while additional signals attributed to monosubstituted anions H_*x*_Na_3–*x*_[SiW_11_MO_39_]^3–^ (M = Co^II^ and Ni^II^, *x* = 1–3) at *m*/*z* 912.1, 919.4, and 926.8 for the Co^II^ representative and at *m*/*z* 912.0, 919.3, and 926.7 for the Ni^II^ representative were detected in 100 mM NaOAc/AcOH (pH 5.5; Figures S13D and S14D). This is another indication of how a buffer affects the POM chemistry that is often overlooked.^20^ Thus, ESI-MS studies indicate probable complete dissociation of the sandwich-type **Na{SiW**_**10**_**M**_**2**_**}**_**2**_ to the disubstituted monomeric species **{SiW**_**10**_**M**_**2**_**}**, followed by further rearrangement to the monosubstituted Keggin representatives **α-{SiW**_**11**_**M}** (M = Co^II^ and Ni^II^) in acetate buffer over 1 week (Figure 1B,C and Scheme S1). Unfortunately, a high concentration of acetate, even in aqueous solutions of **{SiW**_**9**_**Co**_**4**_**}**, interfered with the obtainment of a reasonable mass spectrum because of suppression of the POT signals by signals from acetate complexes. Nevertheless, UV/vis studies (Figure S10B) clearly indicate the rearrangement of **{SiW**_**9**_**Co**_**4**_**}**.

Co-crystallization of proteinase K and the three Keggin POTs **Na{SiW**_**10**_**Co**_**2**_**}**_**2**_, **Na{SiW**_**10**_**Ni**_**2**_**}**_**2**_, and **{SiW**_**9**_**Co**_**4**_**}** was applied. Hanging-drop vapor diffusion in acetate buffer (pH 5.5) yielded high-resolution crystals (Table S6). The protein crystal structures revealed the monosubstituted α-Keggin POTs (Figure S17) α-[SiW_11_O_39_{Co(H_2_O)}]^6–^ (**α-{SiW**_**11**_**Co}**) and α-[SiW_11_O_39_{Ni(H_2_O)}]^6–^ (**α-{SiW**_**11**_**Ni}**) localized in the same two interaction sites on the protein surface and in identical orientations as observed before^17^ (position 1, Figure 2, and position 2, Figure 3) when applying **Na{SiW**_**10**_**M**_**2**_**}**_**2**_ and **{SiW**_**9**_**Co**_**4**_**}**. As is known for other sandwich POTs,^27^ the monomeric forms (Figure S19) may be intermediately provided by hydrolysis, which was confirmed by ESI-MS for **Na{SiW**_**10**_**Ni**_**2**_**}**_**2**_ and **Na{SiW**_**10**_**Co**_**2**_**}**_**2**_ (Figures S13 and S14 and 1B), thereby leading to better accessibility of the transition-metal sites^28^ with a high tendency to form covalent bonds to protein side chains. The Keggin polyanions are covalently bound to the aspartate D207 carboxylate (average metal–O distance, 1.6 Å; position 1, Figure 2) by their Ni^II^ and Co^II^ centers, with similar bond distances compared to the P-centered Keggin POTs.^17^ The POT in position 1 interacts with two more protein molecules by hydrogen-bonding (Figure S18A), whereas the Keggin binding position 2 (Figure 3) is located in the proximity of serine S45, where the POTs participate in an extended network of hydrogen bonds to mostly main-chain peptide groups. Another protein molecule is coordinated in position 2 from the opposite side of the POT (Figure S18B). A more precise inspection of the protein-bound amounts of **α-{SiW**_**11**_**Co}** and **α-{SiW**_**11**_**Ni}** (in terms of an anomalous signal, see Tables S8 and S9) revealed that **α-{SiW**_**11**_**Co}** showed a higher affinity for protein interaction, independent of its initial POT source. These considerations are in accordance with the hard–soft acid–base concept, where Co^II^ showed a higher affinity to the carboxylate side chain than the softer metal Ni^II^.^17^

**Figure 2:**
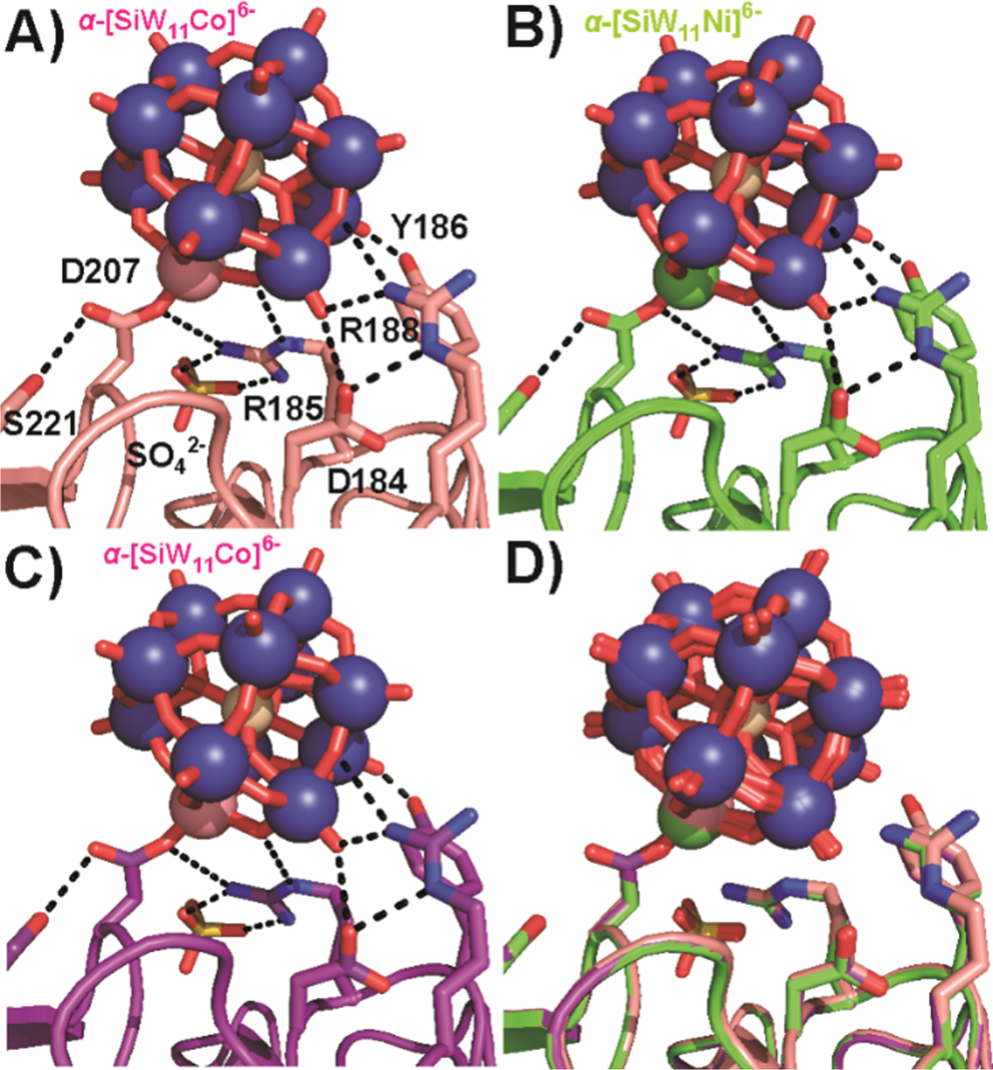
Covalent bond formation of Keggin POTs with aspartate D207 (position 1). R185 coordinating a sulfate anion is involved in strong hydrogen bonds to the POT O atoms. Color code: W, blue; O, red; Si, ivory; Co, rose; Ni, green; N, blue; S, yellow. The interacting side chains are depicted as sticks and interactions as black dashed lines (Table S8). One-letter code for amino acids: D, aspartic acid; R, arginine; S, serine; Y, tyrosine. (A) α-[SiW_11_Co]^6–^, formed from **Na{SiW**_**10**_**Co**_**2**_**}**_**2**_. (B) α-[SiW_11_Ni]^6–^, formed from **Na{SiW**_**10**_**Ni**_**2**_**}**_**2**_. (C) α-[SiW_11_Co]^6–^, formed from **{SiW**_**9**_**Co**_**4**_**}**. (D) Overlay of the three structures (A, rose; B, green; C, purple) for comparison.

**Figure 3:**
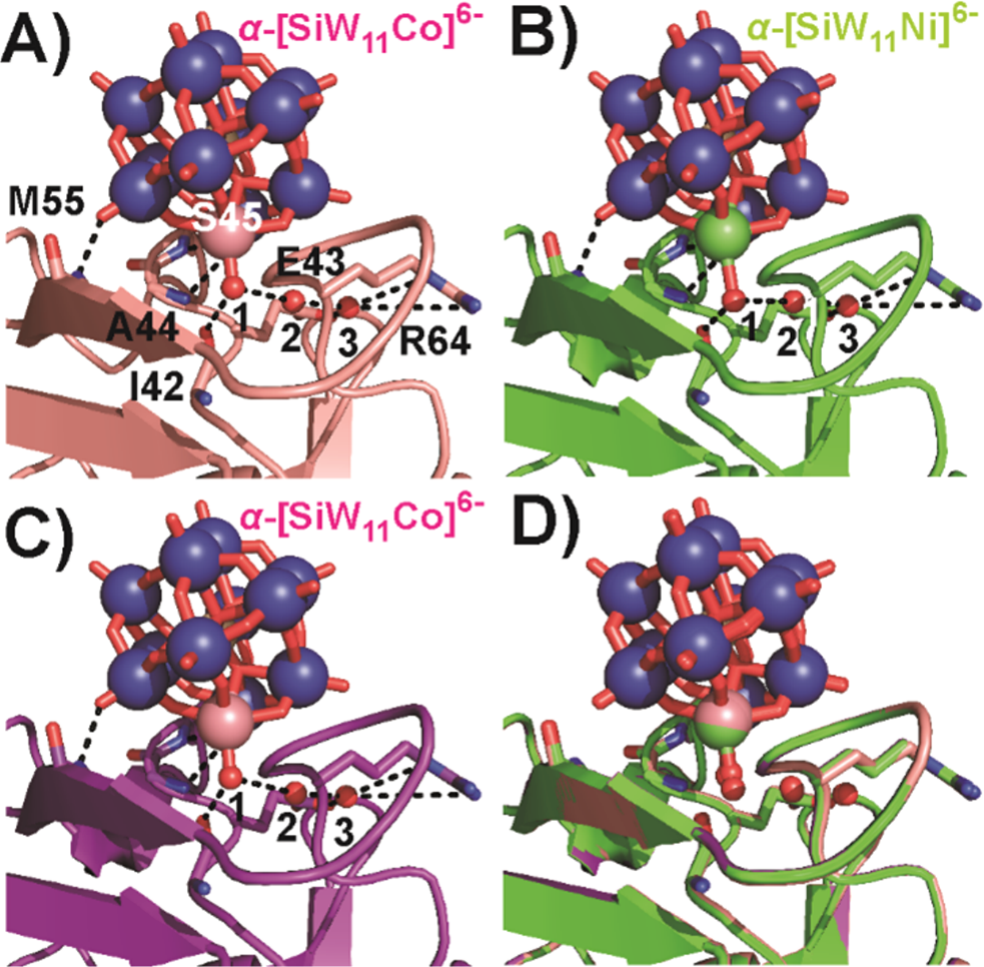
Keggin POT binding position 2. Incorporation of the Keggin POT aquo ligand in the hydrogen network (black dashed lines, Table S9) close to S45. The aquo ligand and other conserved water molecules are numbered by 1–3. The POT interaction is mainly stabilized by hydrogen-bonding to the protein backbone. One-letter code for amino acids: A, alanine; E, glutamic acid; I, isoleucine; M, methionine; R, arginine; S, serine. (A) α-[SiW_11_Co]^6–^, formed from **Na{SiW**_**10**_**Co**_**2**_**}**_**2**_. (B) α-[SiW_11_Ni]^6–^, formed from **Na{SiW**_**10**_**Ni**_**2**_**}**_**2**_. (C) α-[SiW_11_Co]^6–^, formed from **{SiW**_**9**_**Co**_**4**_**}**. (D) Overlay of selected structures (A, rose; B, green; C, purple) for comparison.

Between the two identified POT species that are present in the crystallization cocktail according to ESI-MS in acetate buffer at pH 5.5, only proteinase K crystals with monosubstituted anions were detected, which may have its origin in the different charge densities of both POT species. **{SiW**_**10**_**Co**_**2**_**}** and **{SiW**_**10**_**Ni**_**2**_**}** feature a charge density of 8:12 = 0.67, and **{SiW**_**9**_**Co**_**4**_**}** (Figure S5C) gives a value of 8:13 = 0.61, whereas the monosubstituted **α-{SiW**_**11**_**Co}** and **α-{SiW**_**11**_**Ni}** (Figure S19) show a reduced charge density of 6:12 = 0.5 (Table S7), which is closer to the value 5:12 = 0.42 of the P-centered compounds previously analyzed.^17^

Recently, it was shown that the affinity of POMs toward biomolecules is attributable to their superchaotropic character,^29,30^ and POMs with moderate charge densities (*q*/*m* = 0.33–0.5) interact considerably strongly with surfaces of different or mixed polarities, which are present in proteins.^30−32^ A similar effect was observed for Wells–Dawson-type sandwich anions and predicted for Keggin POTs as well.^33^ Therefore, these binding sites obviously provide a chemical environment of balanced surface polarity (Figure S17) with pronounced specificity for monomeric Keggin POT anions of suitable surface charge density.

In conclusion, the Na salts of the tetrasubstituted Keggin POTs **Na{SiW**_**10**_**Co**_**2**_**}**_**2**_ and **Na{SiW**_**10**_**Ni**_**2**_**}**_**2**_ were synthesized using a new synthesis protocol and studied along with the tetrasubstituted monomeric analogue **{SiW**_**9**_**Co**_**4**_**}** toward their potential as protein crystallization additives. Time-dependent UV/vis spectroscopy and ESI-MS speciation studies under crystallization conditions (acetate buffer, pH 5.5) showed that after 1 week aging mono- and disubstituted Keggin POTs are the predominant species in solution. X-ray crystallographic investigations on the protein crystal structures revealed only the monosubstituted Keggin monomers **α-{SiW**_**11**_**Co}** and **α-{SiW**_**11**_**Ni}**. The selective binding of proteinase K to the monosubstituted anions is explained by their preferable charge density. These findings underline the importance of speciation studies when POTs are applied in solution.

## Supporting Information Available

Details of syntheses, IR, TGA, SXRD, PXRD, UV/vis spectroscopy, ESI-MS, and protein crystallization (PDF)

## Accession Codes

CCDC 2039844 and 2039852 contain the supplementary crystallographic data for this paper. These data can be obtained free of charge via www.ccdc.cam.ac.uk/data_request/cif, or by emailing data_request@ccdc.cam.ac.uk, or by contacting The Cambridge Crystallographic Data Centre, 12 Union Road, Cambridge CB2 1EZ, UK; fax: +44 1223 336033. The crystal structures of proteins obtained in this study are available from the Protein Data Bank (http://www.rcsb.org) as PDB entries 7A9F, 7A9K, and 7A9M.
