# Supplementary material for: Speciation of Transition-Metal-Substituted Keggin-Type Silicotungstates Affected by the Co-crystallization Conditions with Proteinase K
Source: Inorg Chem. 2021 Sep 16;60(20):15096–100. doi: 10.1021/acs.inorgchem.1c02005 (PMC8527451; doi:10.1021/acs.inorgchem.1c02005)
Supplement: Supplementary file 1 — ic1c02005_si_001.pdf [file ic1c02005_si_001.pdf]

ELECTRONIC SUPPLEMENTARY INFORMATION

Speciation of Transition-Metal-Substituted Keggin-Type Silicotungstates Affected by the Co-crystallization Conditions with Proteinase K

Joscha Breibeck[†,+], Elias Tanuhadi[†,+], Nadiia I. Gumerova[†], Gerald Giester[‖], Alexander Prado-Roller[‡], and Annette Rompel[*,†]

[†] Universität Wien, Fakultät für Chemie, Institut für Biophysikalische Chemie, Althanstr. 14, 1090 Wien, Austria. www.bpc.univie.ac.at

[‖] Universität Wien, Fakultät für Geowissenschaften, Geographie und Astronomie, Institut für Mineralogie und Kristallographie, Althanstr. 14, 1090 Wien, Austria.

[‡] Universität Wien, Fakultät für Chemie, Institut für Anorganische Chemie und Zentrum für Röntgenstrukturanalyse, Währinger Str. 42, 1090 Wien, Austria.

[+] These authors contributed equally to this work.

* correspondence to: annette.rompel@univie.ac.at

Content

1. General Information 3
2. Synthesis of polyoxotungstates..... 5
2.1. Preparation of $Na_{14}[(A\text{-}\alpha\text{-}SiW_{10}O_{37})_2\{Co_4(OH)_2(H_2O)_2\}] \cdot 37\ H_2O$ **Na{SiW$_{10}$Co$_2$}$_2$** 5
2.2. Preparation of $Na_{14}[(A\text{-}\alpha\text{-}SiW_{10}O_{37})_2\{Ni_4(OH)_2(H_2O)_2\}] \cdot 77.5\ H_2O$ **Na{SiW$_{10}$Ni$_2$}$_2$**..... 5
2.3. Preparation of $K_5Na_3[A\text{-}\alpha\text{-}SiW_9O_{34}(OH)_3\{Co_4(OAc)_3\}] \cdot 18\ H_2O$ **{SiW$_9$Co$_4$}**..... 5
3. IR-spectra 6
4. Thermogravimetric analysis..... 8
5. Single-Crystal X-ray Diffraction 10
6. Powder X-ray Diffraction 12
7. UV/Vis Spectroscopy 14
8. ESI MS 19
9. Polyoxotungstate - Proteinase K co-crystallization..... 21
9.1. Preparation of Proteinase K for crystallization..... 21
9.2. Preparation of Polyoxotungstate-Proteinase K co-crystals 23
9.3. X-ray data collection, structure solution and refinement..... 24
9.4. Binding positions of Keggin anions..... 25
10. References 30

1. General Information

All reagents and chemicals were of high-purity grade and were used as purchased without further purification. $Na_{10}[A\text{-}\alpha\text{-}SiW_9O_{34}]$ was prepared according to the literature procedure.[1]

Elemental analysis was carried out in aqueous solutions with 2 % HNO_3 employing inductive-coupled plasma mass spectrometry (PerkinElmer Elan 6000 ICP-MS) and atomic absorption spectroscopy (PerkinElmer 1100 Flame AAS). Standards (Merck, Ultra Scientific and Analytika Prague) were prepared from 1000 mg/L single-element standard solutions.

Attenuated total reflection Fourier−transform Infrared Spectroscopy (ATR FTIR): All spectra were recorded on a Bruker Vertex70 IR Spectrometer equipped with a single−reflection diamond−ATR unit. Frequencies are given in cm^{-1}, intensities denoted as w = weak, m = medium, s = strong.

Thermogravimetric analysis (TGA): TGA was performed on a Mettler SDTA851e Thermogravimetric Analyzer under N_2 flow with a heating rate of 5 K min^{-1} in the region 298−973 K.

Single crystal X-ray diffraction (SXRD): The X-ray data were measured on a Nonius Kappa-CCD diffractometer, equipped with a 0.3 mm monocapillary optics collimator, graphite monochromatized MoK_α-radiation, at 200 K (**Na{SiW$_{10}$Co$_2$}$_2$** and **Na{SiW$_{10}$Ni$_2$}$_2$**) and on a Bruker D8 VENTURE equipped with a multilayer monochromator, Mo K_α Incoatec Microfocus sealed tube, and Kryoflex cooling device at 140 K (**{SiW$_9$Co$_4$}**). The structures were solved by direct methods and refined by full-matrix least-squares. Non-hydrogen atoms were refined with anisotropic displacement parameters. The following software was used for the structure-solving procedure: frame integration, Bruker SAINT software package using a narrow-frame algorithm (absorption correction)[2], SADABS[3], SHELXS-2013[4] (structure solution), SHELXL-2013[5] (refinement), OLEX2[6] (structure solution, refinement, molecular diagrams, and graphical user-interface), and SHELXLE[7] (molecular diagrams and graphical user interface). Experimental data are provided in **Table S4**.

Powder X-ray diffraction was performed on an EMPYREAN diffractometer system using CuK_α radiation (λ = 1.540598), a PIXcel3D-Medipix3 1 × 1 detector (used as a scanning line detector) and a divergence slit fixed at 0.1 mm. The scan range was from 5° to 50° (2θ).

UV/Vis spectroscopy: UV/Vis spectra were collected on a Shimadzu UV 1800 spectrophotometer equipped with a Julaba F25 water bath to ensure a constant temperature of 20 ± 2°C in the sample cuvette, mimicking the temperature of the crystallization experiments.

Electrospray ionization mass spectrometry (ESI MS): ESI MS was performed with an ESI−Qq−oaRTOF supplied by Bruker Daltonics Ltd. Bruker Daltonics Data Analysis software was used for peaks assignment. The measurement was carried out in H_2O and NaOAc / HOAC buffer diluted with 50 % of ACN, collected in negative and positive ion mode and with the spectrometer calibrated with the standard tune−mix to give an accuracy of ca. 5 ppm in the region of m/z 300−1900. The signals with low intensities show an accuracy of ca. 10 ppm.

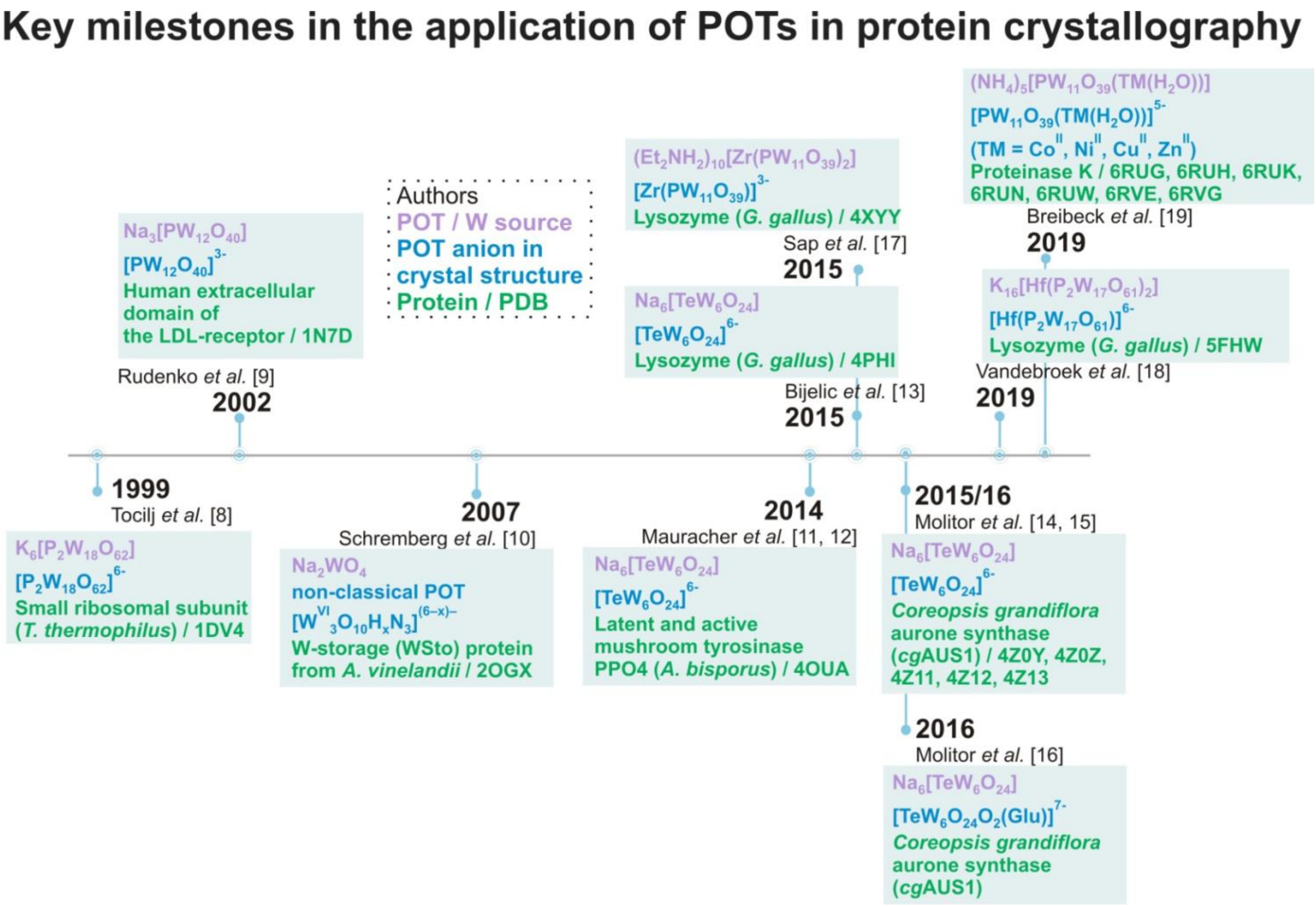

Figure S1. Key milestones in the application of POTs in protein crystallography. For the timeline, investigation, which, in the author's opinion, gave impetus to the further development of the POT's application in protein crystallography, are highlighted [8, 9, 10, 11, 12, 13, 14, 15, 16, 17, 18, 19]. The names of the first authors are shown in black, the solid POT in purple, the POT anion detected in crystal structure in blue, the protein and PDB entries in green.

2. Synthesis of polyoxotungstates

2.1. Preparation of $Na_{14}[(A\text{-}\alpha\text{-}SiW_{10}O_{37})_2\{Co_4(OH)_2(H_2O)_2\}] \cdot 37\ H_2O$ **Na{SiW$_{10}$Co$_2$}$_2$**

To a vigorously stirred solution of $Co(OAc)_2 \cdot 4\ H_2O$ (1.5 g, 6 mmol) in water (75 ml) $Na_{10}[A\text{-}\alpha\text{-}SiW_9O_{34}]$ (5 g, 2 mmol) was added in small portions followed by stepwise heating of the reaction mixture to 80°C. After addition of 50 mL of water and reaction at 80 °C for 1 h, the reaction mixture was cooled to room temperature and passed through a cation-exchange column (AMBERLITE™ IR120 resin, Lenntech) which was equilibrated with water. Addition of a solution of NaCl (1.6 g) in 6.25 mL water to the collected eluate and slow evaporation of the filtered solution at room temperature led to formation of dark red rod-shaped crystals of **Na{SiW$_{10}$Co$_2$}$_2$** after four weeks. Yield: 2.5 g, 20% based on W. Anal. Calcd. (%) for $Na_{14}H_{80}Si_2W_{20}O_{115}Co_4$ ($Na_{14}[(A\text{-}\alpha\text{-}SiW_{10}O_{37})_2\{Co_4(OH)_2(H_2O)_2\}] \cdot 37\ H_2O$): Na, 5.18; Si, 0.90; Co, 3.80. Found: Na, 6.71; Si, 0.87; Co, 2.21.

2.2. Preparation of $Na_{14}[(A\text{-}\alpha\text{-}SiW_{10}O_{37})_2\{Ni_4(OH)_2(H_2O)_2\}] \cdot 77.5\ H_2O$ **Na{SiW$_{10}$Ni$_2$}$_2$**

The synthesis of **Na{SiW$_{10}$Ni$_2$}$_2$** was similar to that of **Na{SiW$_{10}$Co$_2$}$_2$** except that a solution containing 1.5 g of $NiSO_4 \cdot 7\ H_2O$ in 75 mL 0.5 M NaOAc pH 6.5 was applied as transition metal source instead of $Co(OAc)_2 \cdot 4\ H_2O$. Yield: 2.1 g, 15% based on W. Anal. Calcd. (%) for $Na_{14}H_{161}Si_2W_{20}O_{155}Ni_4$ ($Na_{14}[(A\text{-}\alpha\text{-}SiW_{10}O_{37})_2\{Ni_4(OH)_2(H_2O)_2\}] \cdot 77.5\ H_2O$): Na, 4.64; Si, 0.81; Ni, 3.39. Found: Na, 6.11; Si, 0.93; Ni, 3.75.

2.3. Preparation of $K_5Na_3[A\text{-}\alpha\text{-}SiW_9O_{34}(OH)_3\{Co_4(OAc)_3\}] \cdot 18\ H_2O$ **{SiW$_9$Co$_4$}**

The acetato-capped tetra-Co(II) POT **{SiW$_9$Co$_4$}** was synthesized as described by Lisnard *et al.* in [20] and characterized by IR-spectroscopy (**Figure S2**) and SXRD (**Table S4**).

3. IR-spectra

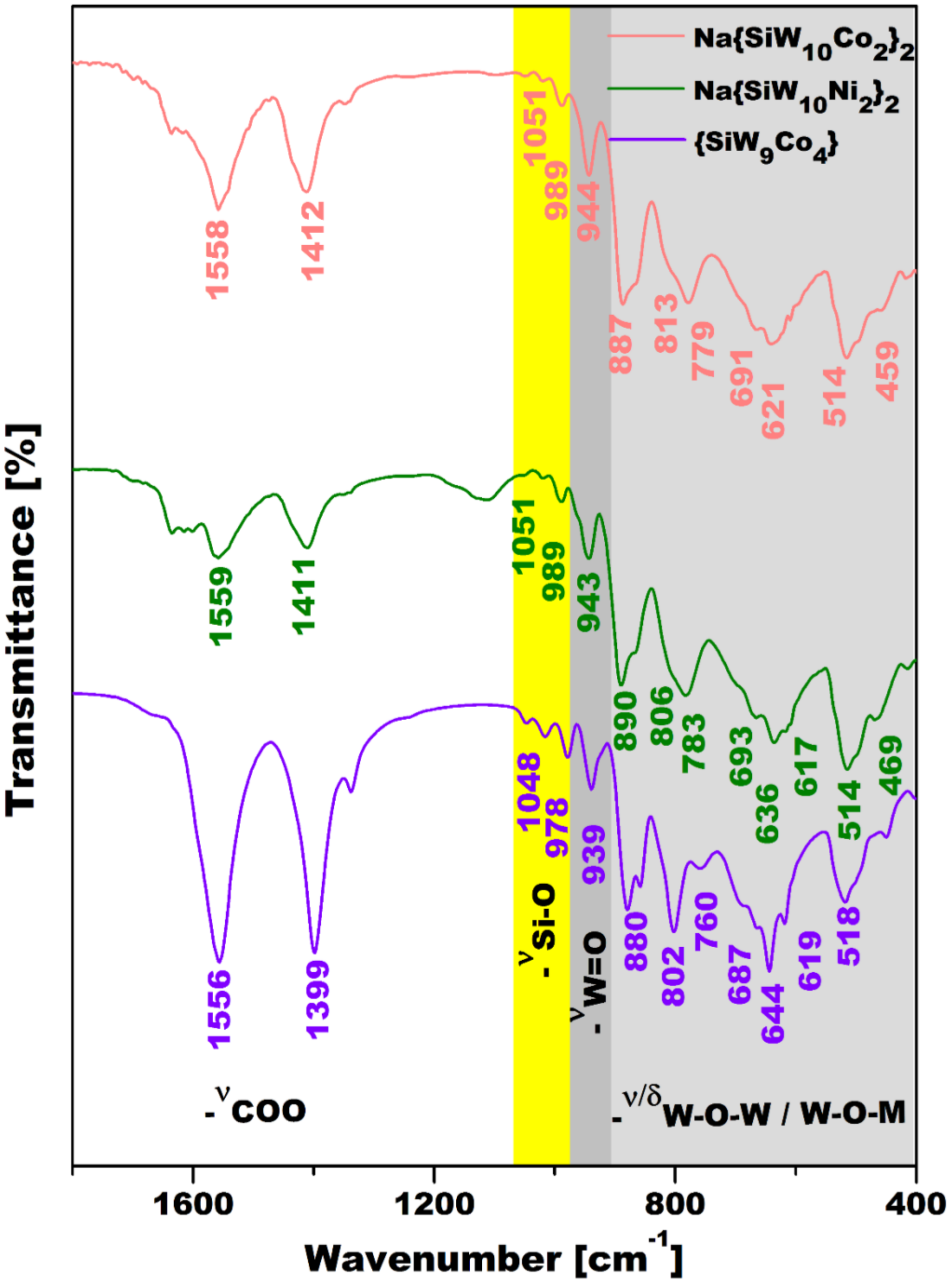

Figure S2. IR analysis of Keggin POT derivatives. The *α*-Keggin Sandwich derivatives **Na{$SiW_{10}Co_2$}$_2$** and **Na{$SiW_{10}Ni_2$}$_2$** feature slight impurities of acetate salt with signals at 1558 and 1412 cm^{-1} (correspond to orange spectra of **Na{$SiW_{10}Co_2$}$_2$**). These signals are strongly pronounced for the acetate-bridged POT **{SiW_9Co_4}**. As can be expected from the shared W-O core structure, the IR spectra are highly similar for all Keggin POT derivatives.[21] For assignment of characteristic vibrations of the Keggin POT scaffold, see **Table S1**. ν: vibration, δ: distortion, M: transition metal.

Table S1. IR analysis of Keggin POT derivatives. Due to their structural similarity, the sandwich-type POT anions yielded strikingly similar signals. All samples gave rise to carboxylate vibrations from acetate, but only the acetate-capped POT showed strong signals of this ligand as part of its structure. The vibrations of COO^- visible in the IR spectra of **Na{SiW$_{10}$Co$_2$}$_2$** and **Na{SiW$_{10}$Ni$_2$}$_2$** indicate the presence of a small amount of simple acetate salt, which was introduced during the synthesis using $Co(OAc)_2$ and $Ni(OAc)_2$ as educts, respectively. v: vibration, δ: distortion, M: transition metal.

POT	Wavenumbers [cm^{-1}]	Wavenumbers [cm^{-1}] for the literature analogues (**{SiW$_9$Co$_4$}**[20] and **CsNa{SiW$_{10}$M}$_2$**[22])	Attribution
**Na{SiW$_{10}$Co$_2$}$_2$**	1558, 1412	No data provided	ν COO^-
	1051, 1020	No data provided	ν Si-O
	989, 944	982, 939	ν W=O
	887, 865	895, 870	ν,δ W-O-W/W-O-Co
	813	799	
	779		
	691, 667	715, 689	
	641		ν,δ W-O-W
	621		
	514, 459	530, 462	ν,δ W-O-W/W-O-Co
**Na{SiW$_{10}$Ni$_2$}$_2$**	1559, 1411	No data provided	ν COO^-
	1051, 1020	No data provided	ν Si-O
	989, 943	987, 942, 938	ν W=O
	890, 867	892, 869	ν,δ W-O-W/W-O-Ni
	806	812	
	783	779	
	693, 667	725, 689,	
	636		ν,δ W-O-W
	617		
	514, 469	529, 462	ν,δ W-O-W/W-O-Ni
**{SiW$_9$Co$_4$}**	1556, 1399	1606, 1556, 1443, 1421, 1348	ν COO^-
	1048, 1016		ν Si-O
	978, 939	983, 944, 933	ν W=O
	880, 859	889, 858	ν,δ W-O-W/W-O-Co
	802	808	
	760	742	
	687, 666	674	
	644		ν,δ W-O-W
	619		
	518	539, 523	ν,δ W-O-W/W-O-Co

4. Thermogravimetric analysis

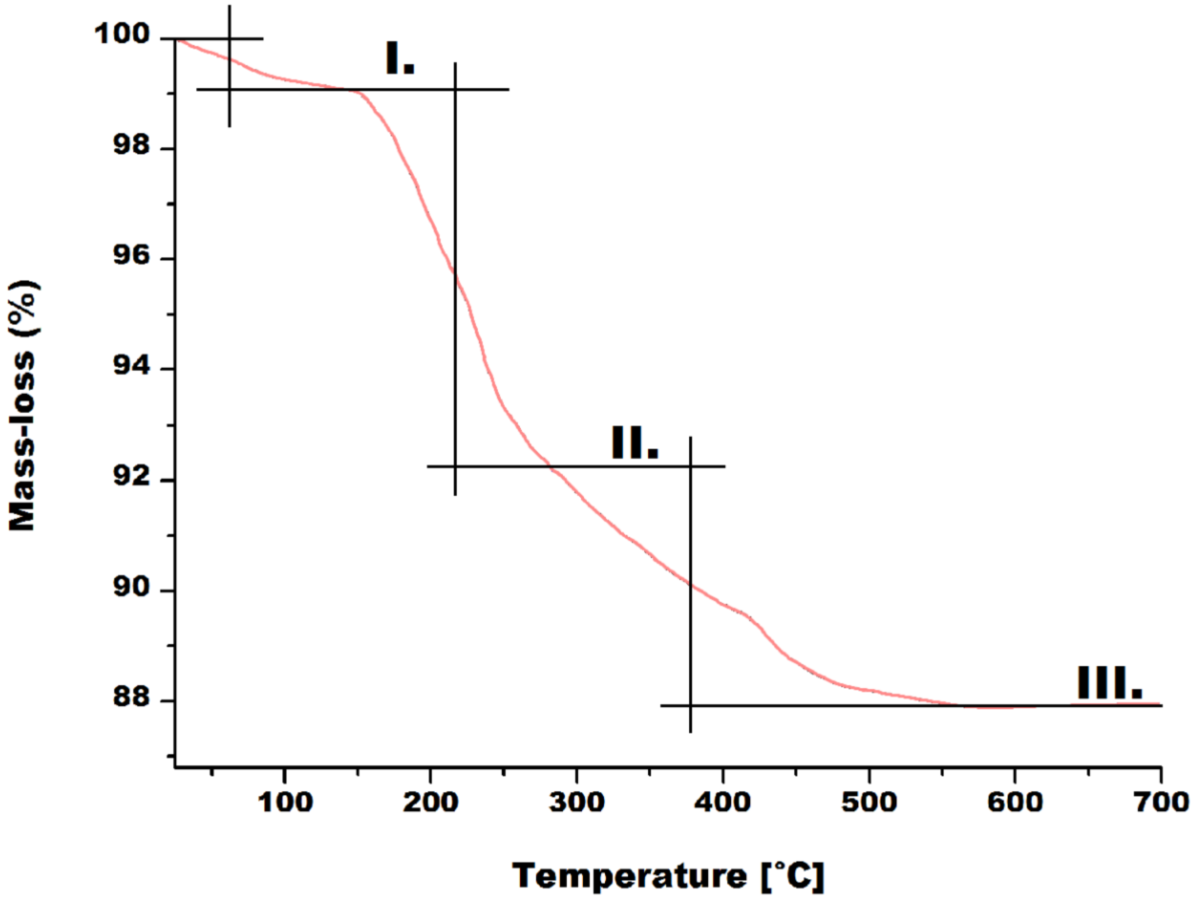

Figure S3. Thermogravimetric curve of $Na_{14}[(\alpha\text{-}SiW_{10}O_{37})_2\{Co_4(OH)_2(H_2O)_2\}]$ • 37 H_2O **Na{SiW$_{10}$Co$_2$}$_2$**.

Table S2. TGA results for $Na_{14}[(\alpha\text{-}SiW_{10}O_{37})_2\{Co_4(OH)_2(H_2O)_2\}]$ • 37 H_2O **Na{SiW$_{10}$Co$_2$}$_2$**.

Step	T, °C	mass-loss, %	number of H_2O molecules corresponding to mass-loss
I	25-150	1.04	3
II	150-300	6.64	22
III	300-700	4.16	14

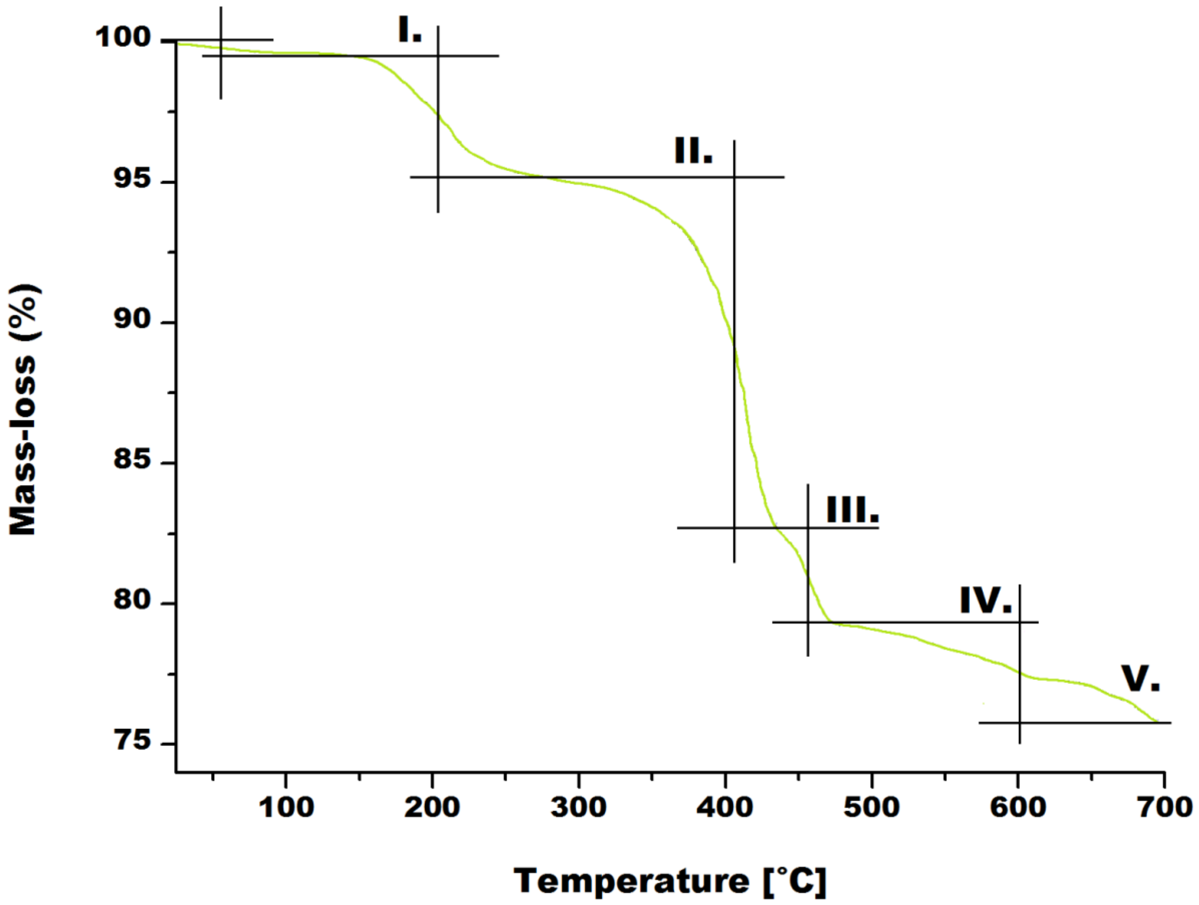

Figure S4. Thermogravimetric curve of $Na_{14}[(\alpha\text{-}SiW_{10}O_{37})_2\{Ni_4(OH)_2(H_2O)_2\}] \cdot 77.5\ H_2O$ **Na{SiW$_{10}$Ni$_2$}$_2$**.

Table S3. TGA results for $Na_{14}[(\alpha\text{-}SiW_{10}O_{37})_2\{Ni_4(OH)_2(H_2O)_2\}] \cdot 77.5\ H_2O$ **Na{SiW$_{10}$Ni$_2$}$_2$**.

Step	T, °C	mass-loss, %	number of H_2O molecules corresponding to mass-loss
I	25-150	0.49	1.5
II	150-250	4.25	14
III	250-430	12.13	42.5
IV	430-470	3.32	10.5
V	470-700	3.46	11

5. Single-Crystal X-ray Diffraction

Table S4. X-ray characteristics of Keggin POT derivatives**.** The full single-crystal X-ray analysis confirmed the successful re-synthesis of **{SiW_9Co_4}**; published cell parameters[20] and temperature are given in brackets for comparison.

abbreviation	**{SiW_9Co_4}**	**Na{$SiW_{10}Co_2$}$_2$**	**Na{$SiW_{10}Ni_2$}$_2$**
**Sum formula**	$C_6H_{48}Co_4K_5Na_3O_{61}SiW_9$	$Na_{14}H_{80}Si_2W_{20}O_{115}Co_4$	$Na_{14}H_{161}Si_2W_{20}O_{155}Ni_4$
**M_t**	3279.3	6211.1	6931.8
**T [K]**	140 (293)	200	200
**Crystal system**	monoclinic	triclinic	triclinic
**Space group**	$P2_1/m$	$P\bar{1}$	$P\bar{1}$
***a* [Å]**	10.7619 (10.7959)	11.4253(19)	11.4457(10)
***b* [Å]**	14.9262 (14.803)	13.697(2)	13.7694(12)
***c* [Å]**	19.141 (19.137)	19.094(3)	19.3056(18)
**α [°]**	90 (90)	89.386(4)	90.966(2)
**β [°]**	93.668 (93.407)	105.382(4)	106.049(2)
**γ [°]**	90 (90)	113.621(4)	112.919(2)
**V [Å^3]**	3069 (3053)	2624.1(7)	2666.4(4)
Z	2 (3)	1	1
**ρ_{calc} [g cm^{-3}]**	-	3.863	3.891
**μ [mm^{-1}]**	-	22.620	22.356
**data/parameters**	-	9498/700	20293/746
**R_{int}**	-	0.0699	0.0759
**goodness of fit**	-	1.048	1.115
**R [I > 2σ(I)]**	-	R_1 = 0.1125<br>wR_2 = 0.2860	R_1 = 0.0458<br>wR_2 = 0.1139

Table S5. Selected bond valence sum (BVS) values for **Na{$SiW_{10}Co_2$}$_2$** and **Na{$SiW_{10}Ni_2$}$_2$**.

POT	Atom sort	Atom center	BVS value
**Na{$SiW_{10}Co_2$}$_2$**	μ_2 - oxo	O28	-2,01
	μ_2 - oxo	O29	-1,87
	μ_2 - oxo	O5	-1,80
	μ_2 - oxo	O37	-1,77
	μ_2 - oxo	O15	-1,74
	μ_2 - oxo	O34	-1,73
	μ_3 - hydroxo	O39	-0,76
**Na{$SiW_{10}Ni_2$}$_2$**	μ_2 - oxo	O29	-1,88
	μ_2 - oxo	O34	-1,83
	μ_2 - oxo	O37	-1,82
	μ_2 - oxo	O5	-1,81
	μ_2 - oxo	O28	-1,80
	μ_2 - oxo	O15	-1,75
	μ_3 - hydroxo	O39	-0,74

The architecture of the isostructural **Na{SiW$_{10}$Ni$_2$}$_2$** and **Na{SiW$_{10}$Co$_2$}$_2$** represents a dimer of [α-SiW$_{10}$O$_{37}$]$^{10-}$ ions (**Figure S5 A, B**). The overall atom connectivity in **Na{SiW$_{10}$M$_2$}$_2$** features a central square-shaped motif of two M^{II} centers and two μ_3-OH. Thereby, all four M^{II} atoms are linked through only two μ_3-oxygens. Since the half-units are related by an inversion center, there are only two structurally non-equivalent types of M^{II} centers exhibiting a distorted octahedral coordination environment with M-O distances ranging from 2.001(6) to 2.27(3) Å, which is in accordance with the findings reported by Haider *et al.*[22] In **Na{SiW$_{10}$M$_2$}$_2$**, each α-SiW$_{10}$O$_{37}$ unit bears one labile water ligand and a very reactive μ_3-OH site, as well as six basic μ_2-O positions bridging W^{VI} and Co^{II} or Ni^{II}. These oxo-sites show a pronounced protonation propensity as indicated by bond valence sum calculations (BVS) (**Table S5**).

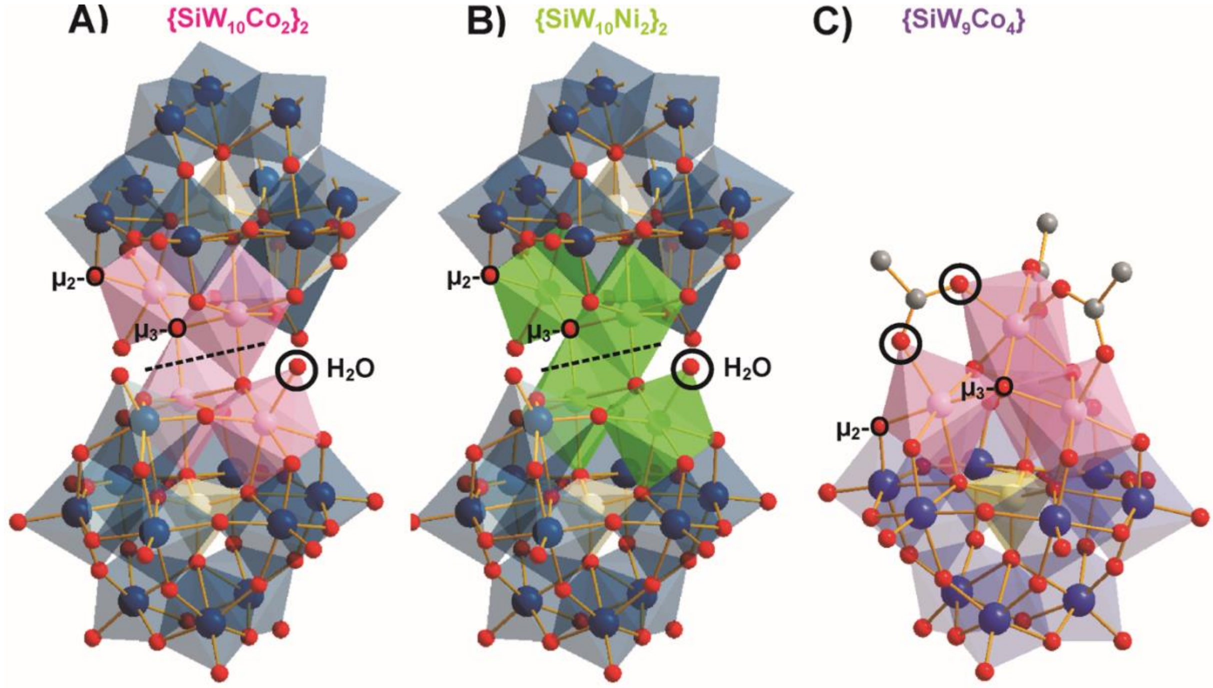

Figure S5. Structures of Keggin POT derivatives **Na{SiW$_{10}$Co$_2$}$_2$ (A)**, **Na{SiW$_{10}$Ni$_2$}$_2$ (B)**, **{SiW$_9$Co$_4$} (C)** in the solid state applied for co-crystallization with proteinase K. Color code: dark blue, W; red, O; rose, Co; green, Ni; ivory, Si; grey, C. All POT structures feature labile aquo or acetato ligands (marked by circles) as well as highly nucleophilic and basic bridging oxo-sites.

6. Powder X-ray Diffraction

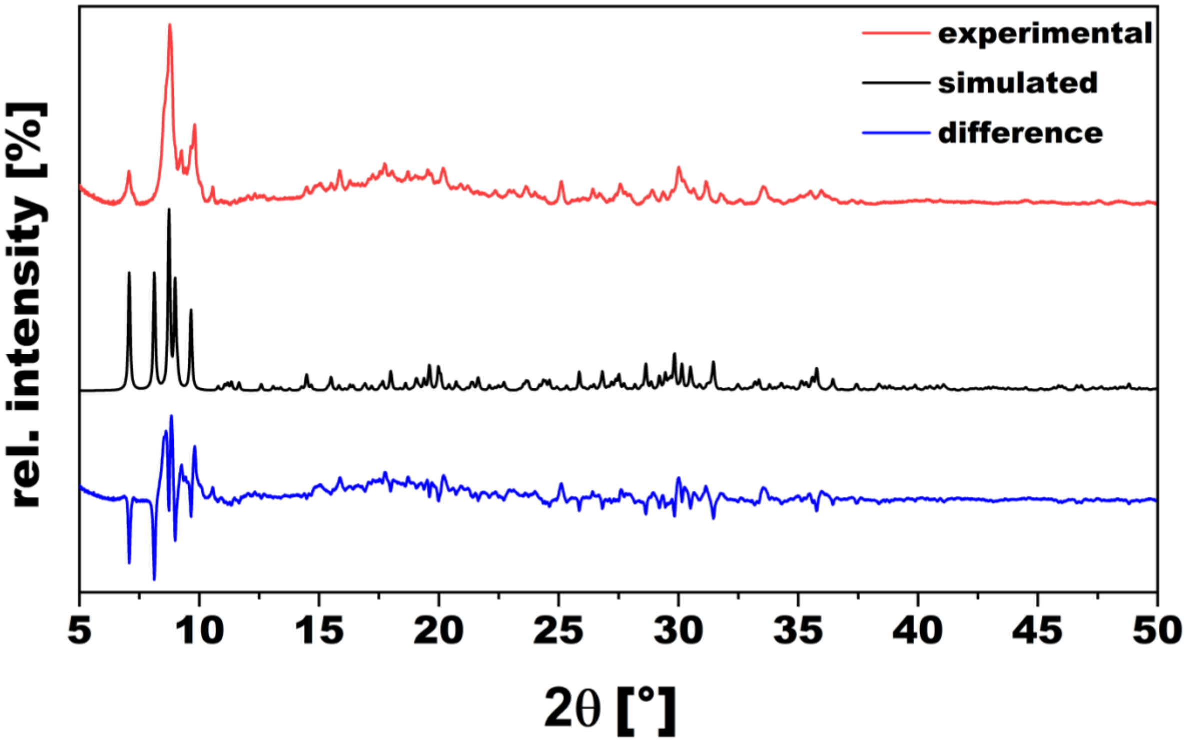

Figure S6. Comparison of the experimental and simulated PXRD patterns of Na_{14}[(*A*-*α*-$SiW_{10}O_{37})_2\{Co_4(OH)_2(H_2O)_2\}$] • 37 H_2O **Na{SiW$_{10}Co_2$}$_2$**. Given the loss of crystal water already occurring at room temperature (please see **Figure S3**, along with **Table S2**), the presence of more than one crystalline phase belonging to the investigated POTs can be suggested, which gave rise to the observed difference in the experimental and simulated patterns.

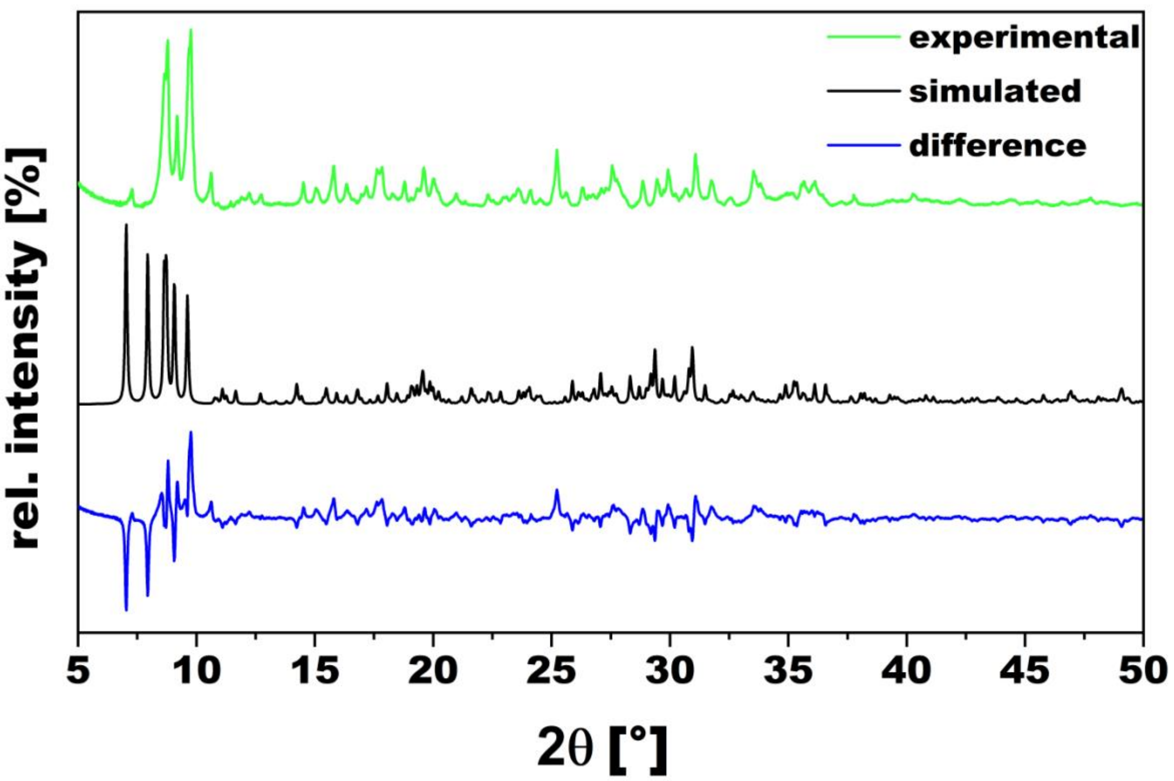

Figure S7. Comparison of the experimental and simulated PXRD patterns of Na_{14}[(*A*-*α*-$SiW_{10}O_{37})_2\{Ni_4(OH)_2(H_2O)_2\}$] • 77.5 H_2O **Na{SiW$_{10}Ni_2$}$_2$**. Given the loss of crystal water already occurring at room temperature (please see **Figure S4**, along with **Table S3**), the presence of more than one crystalline phase belonging to the investigated POTs can be suggested, which gave rise to the observed difference in the experimental and simulated patterns.

7. UV/Vis Spectroscopy

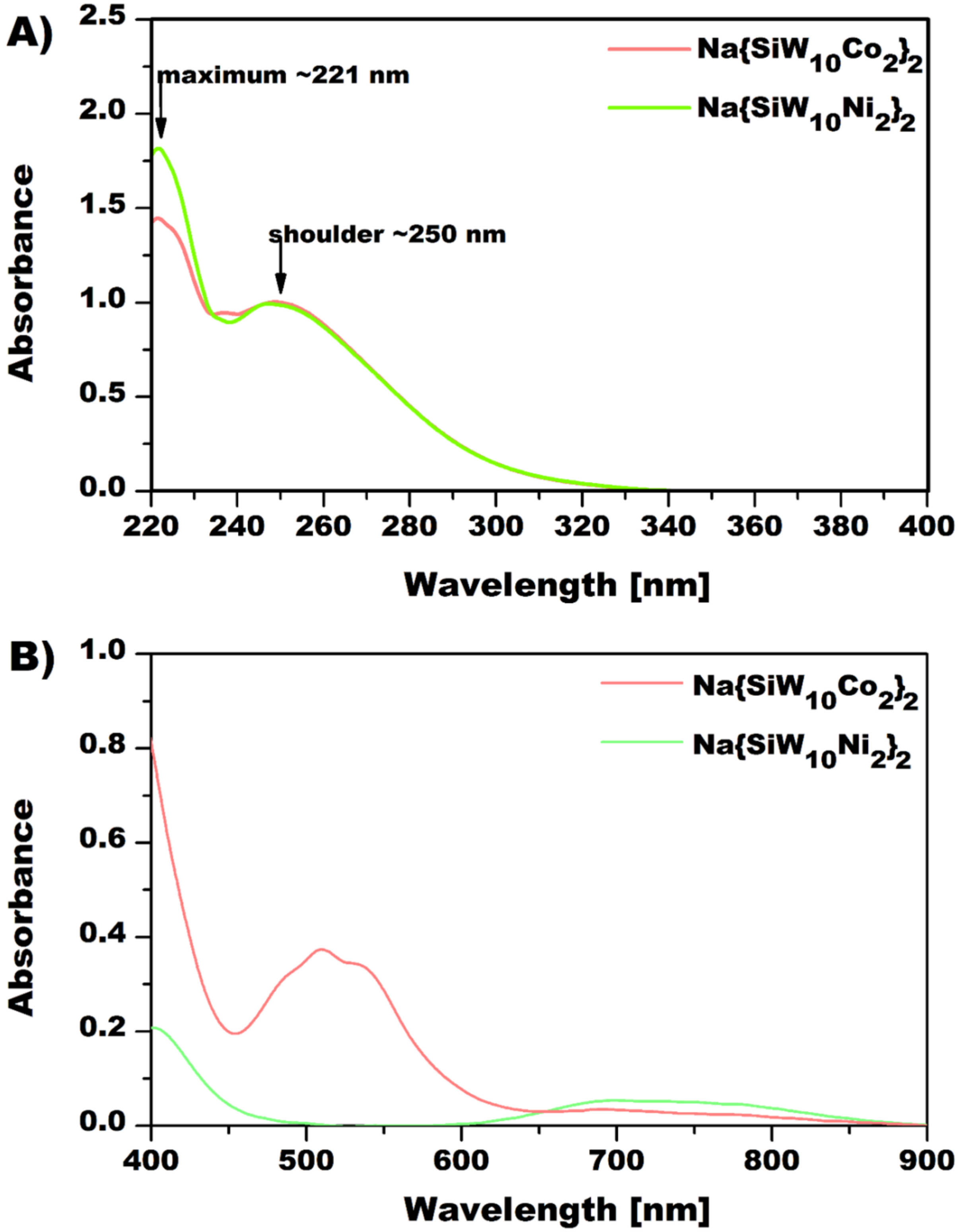

Figure S8. UV/Vis-spectra of **Na{SiW$_{10}$Co$_2$}** and **Na{SiW$_{10}$Ni$_2$}** in H_2O (pH = 6.8 via HCl [1 M]) showing the **A)** typical O→W ligand−to−metal charge−transfer with absorption maximum at ~221 nm and a shoulder at ~250 nm [10 µM] and **B)** the d-d transitions[22] in the visible-NIR region [50 µM].

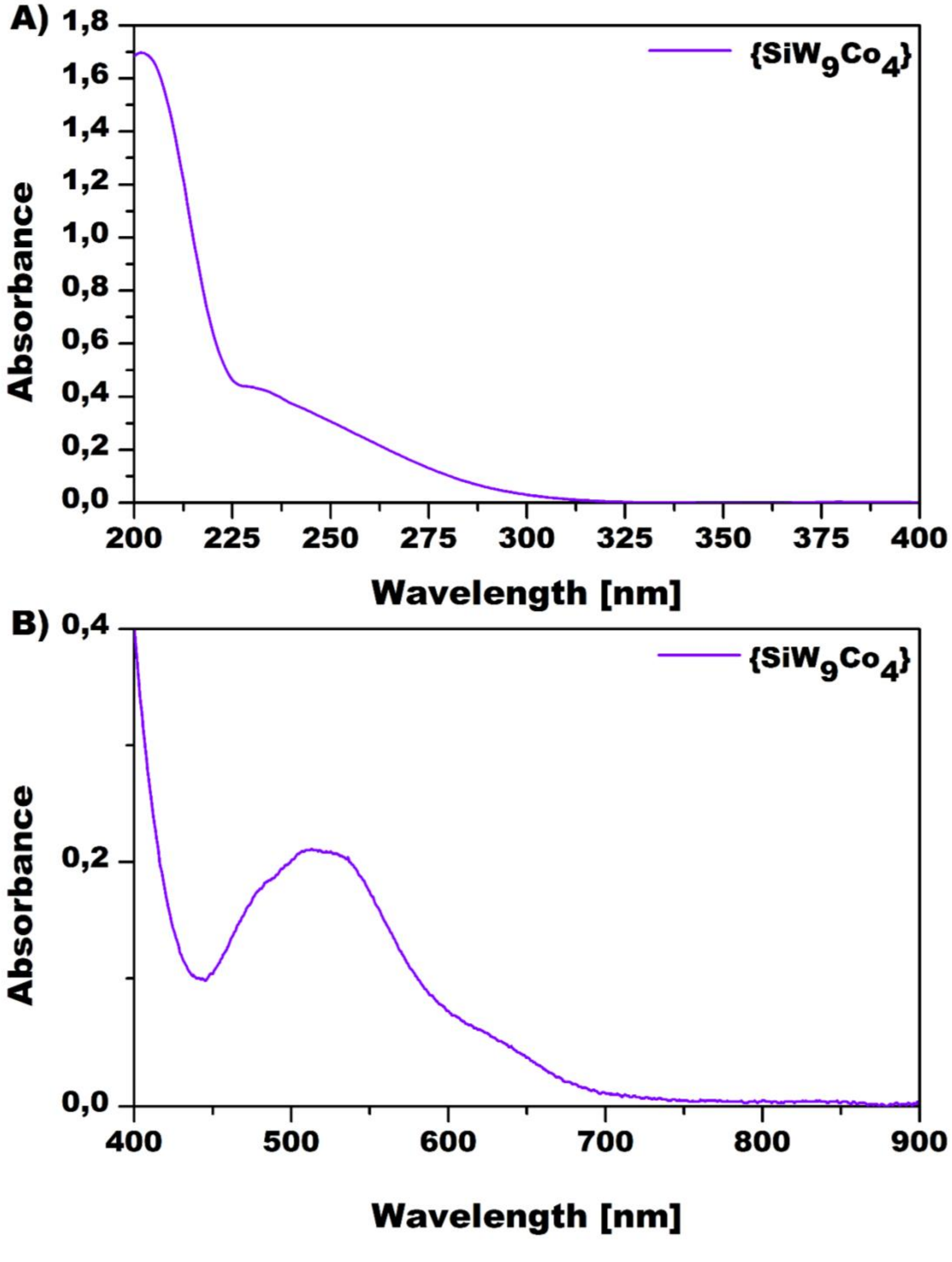

Figure S9. UV/Vis-spectra of **{SiW_9Co_4}** in 1 M NaOAc/AcOH pH 6.0 showing the **A)** typical O→W ligand−to−metal charge−transfer with absorption maximum at ~201 nm and a shoulder at ~250 nm ([12 µM]) and **B)** the d-d transitions[22] in the visible-NIR region ([40 µM]).

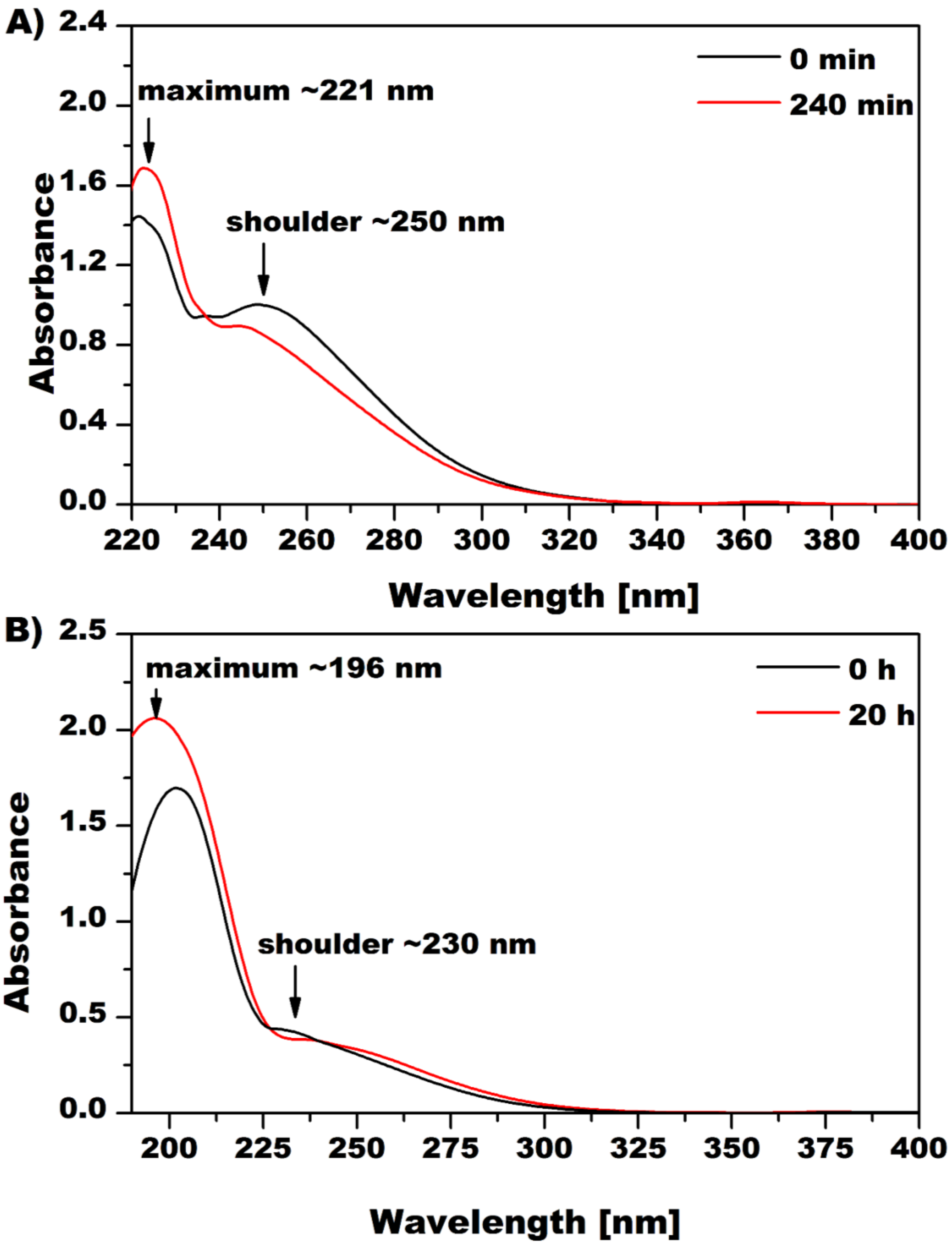

Figure S10. Time-dependent UV/Vis-spectra showing the decreasing shoulder at ~250 nm in the UV/Vis range of **A) Na{$SiW_{10}Co_2$}$_2$** [10 µM] and **B) {SiW_9Co_4}** [12 µM] indicating a rearrangement of the POT framework in 100 mM NaOAc/AcOH pH 5.5.

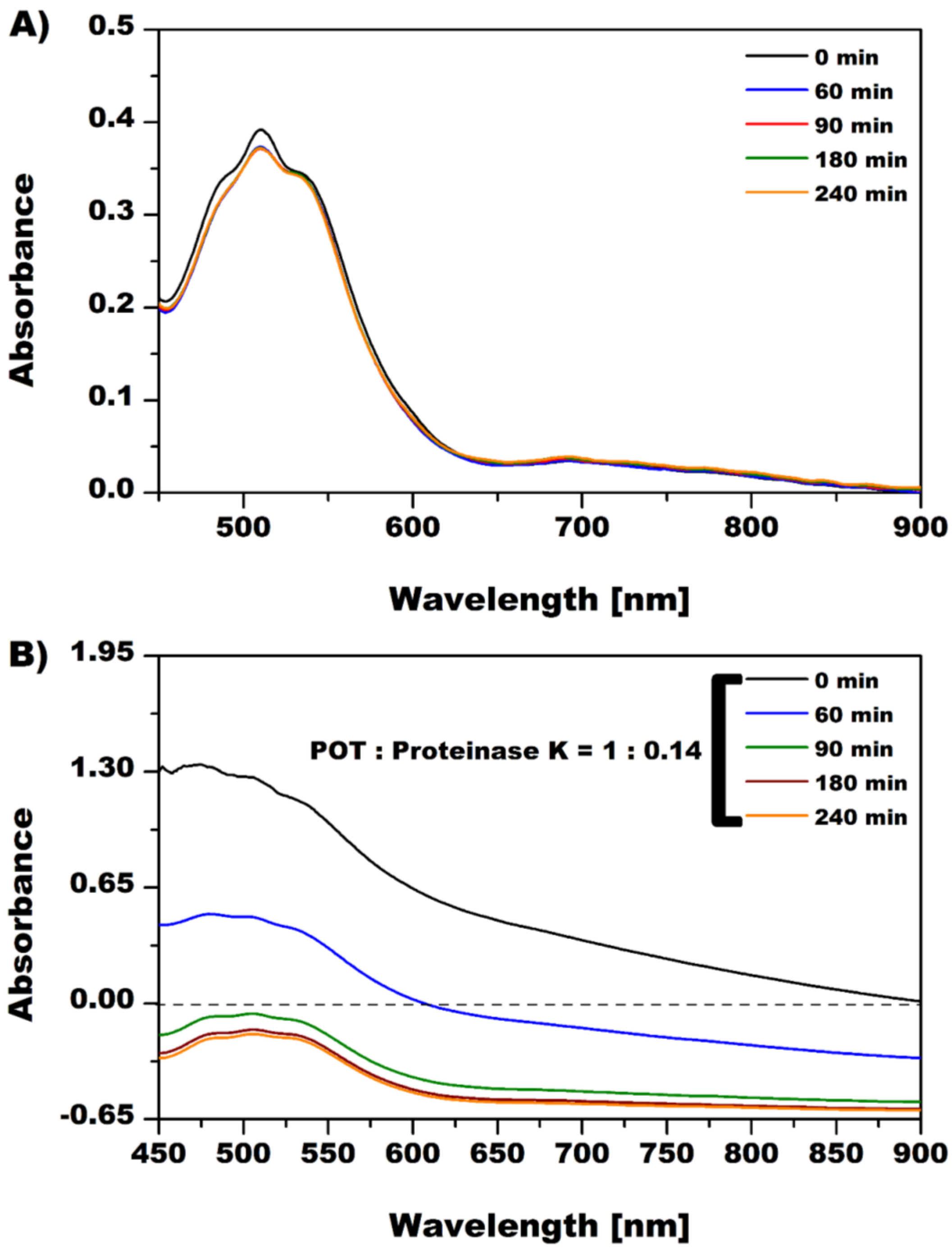

Figure S11. Time-dependent UV/Vis-spectra showing the UV/Vis NIR range of **Na{SiW$_{10}$Co$_2$}$_2$** [50 µM] in the **A)** absence of proteinase K in H_2O (pH = 6.8) and the **B)** presence of proteinase K. The ratio 1:0.14 was chosen to reduce formation of POM – protein precipitates arising from the strong interactions. While the d-d transition band at ~512 nm corresponding to the octahedrally coordinated Co(II) metal centers in **Na{SiW$_{10}$Co$_2$}$_2$** remain almost unchanged over a time period of 240 min, a drastic change of the absorption can be observed in the presence of the protein suggesting immediate POT-protein interaction resulting in adduct formation and precipitation as shown by a substantial decrease in absorption leading to negative values (dotted line).

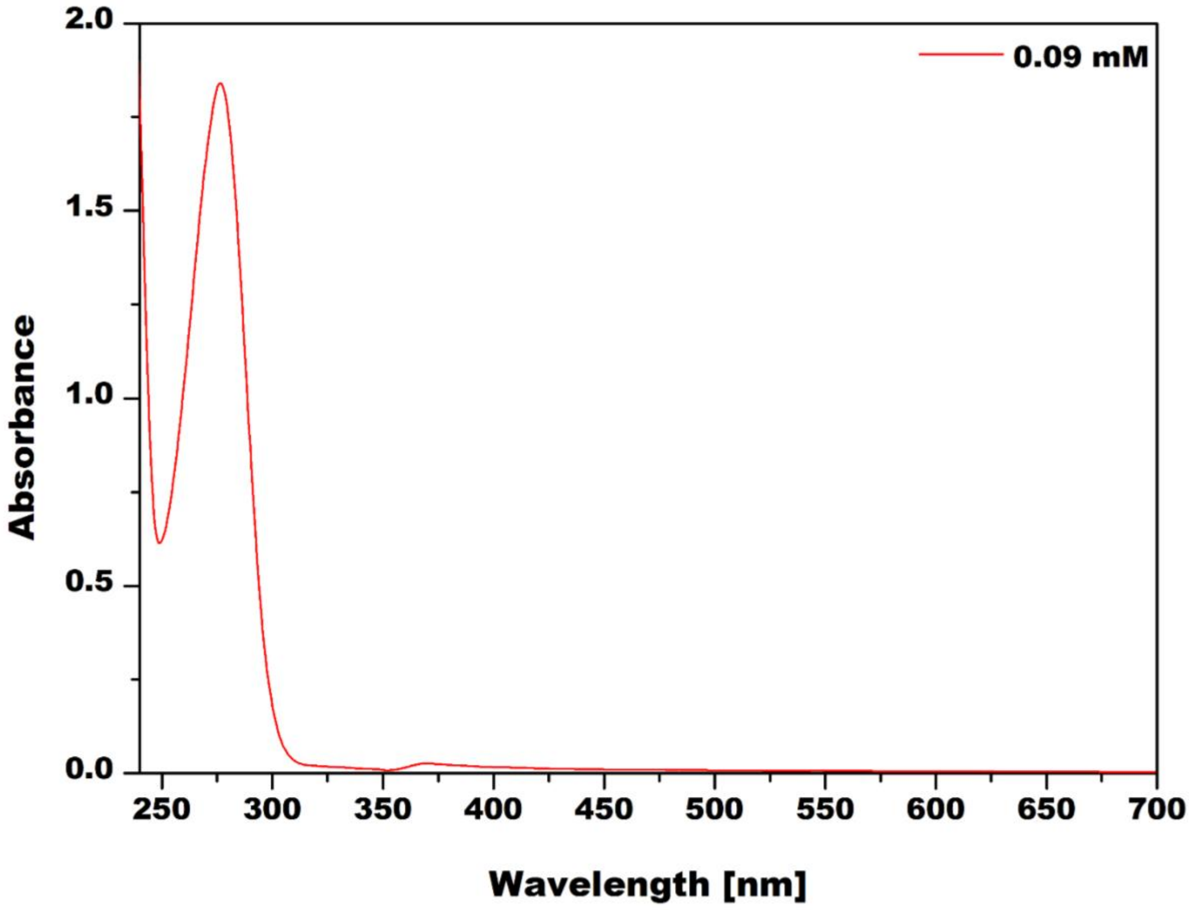

Figure S12. UV/Vis spectrum of proteinase K in 10 mM Tris/HCl pH 7.0 showing the pronounced band at 276 nm attributed to the presence of tyrosine side chains.[23]

8. ESI MS

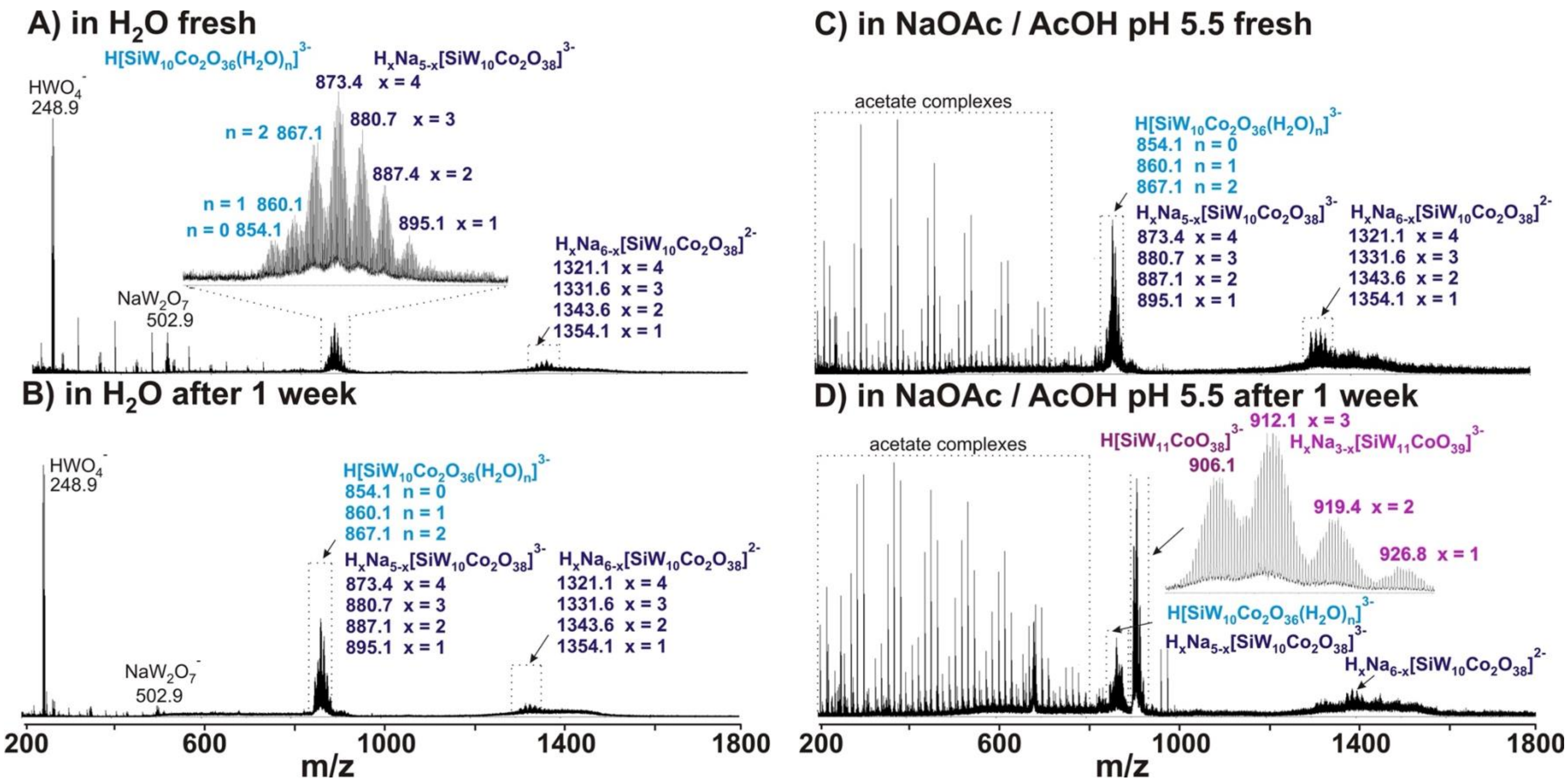

Figure S13. ESI mass spectrum of **Na{$SiW_{10}Co_2$}$_2$** recorded in negative mode in the range 200 to 1800 m/z **A)** in a freshly prepared and **B)** in an aqueous solution aged for one week at room temperature; **C)** in a freshly prepared and **D)** in a sodium acetate buffer (pH 5.5) solution aged for one week at room temperature; for anions $H[SiW_{10}Co_2O_{36}(H_2O)_n]^{3-}$, $H_xNa_{5-x}[SiW_{10}Co_2O_{38}]^{3-}$ and $H_xNa_{6-x}[SiW_{10}Co_2O_{38}]^{2-}$ values n and x are the same as in **A) – C)**.

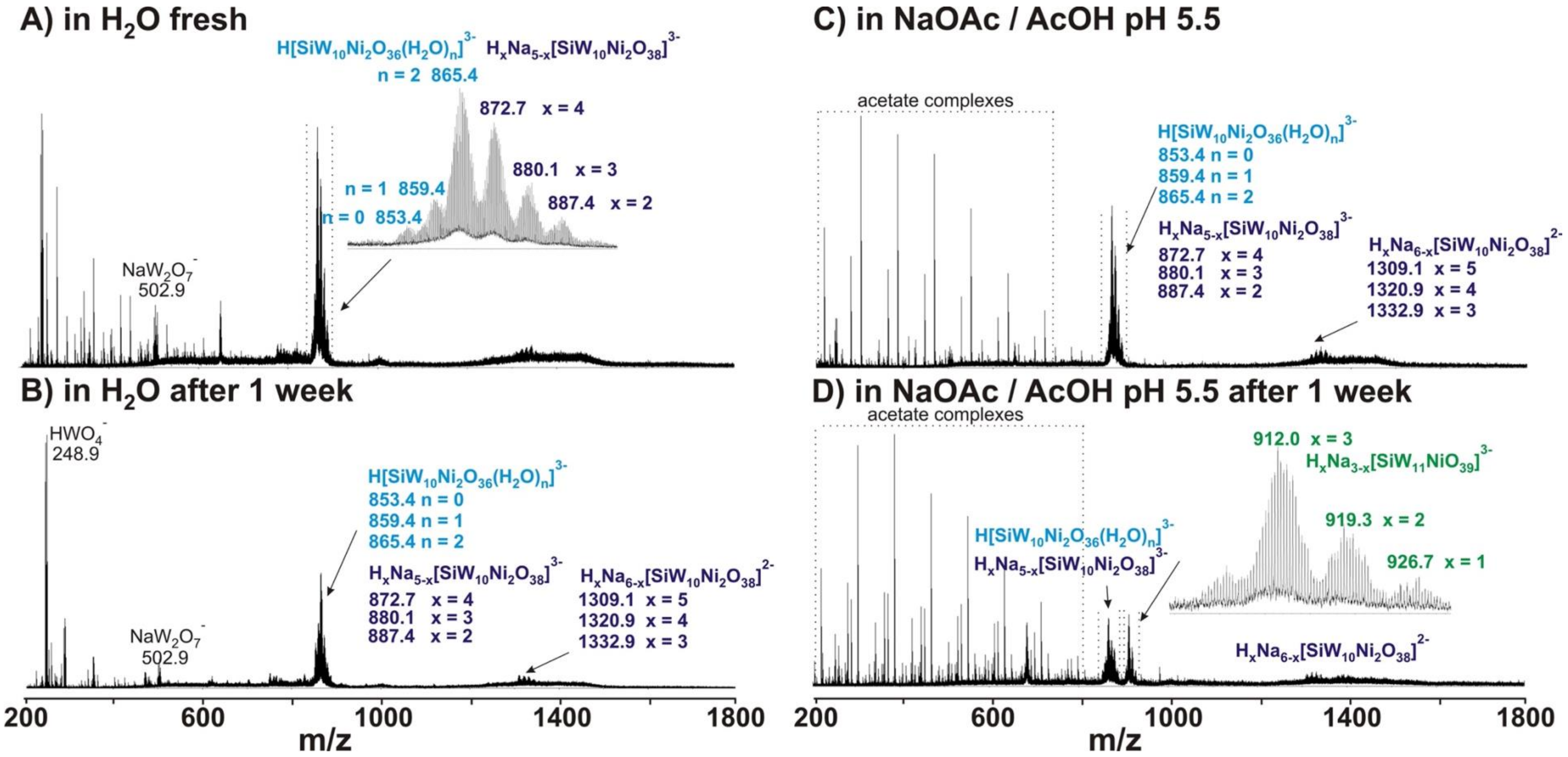

Figure S14. ESI mass spectrum of **Na{SiW$_{10}$Ni$_2$}$_2$** recorded in negative mode in the range 200 to 1800 m/z **A)** in a freshly prepared and **B)** in an aqueous solution aged for one week at room temperature; **C)** in a freshly prepared and **D)** in a sodium acetate buffer (pH 5.5) solution aged for one week at room temperature, for anions $H[SiW_{10}Ni_2O_{36}(H_2O)_n]^{3-}$, $H_xNa_{5-x}[SiW_{10}Ni_2O_{38}]^{3-}$ and $H_xNa_{6-x}[SiW_{10}Ni_2O_{38}]^{2-}$ values n and x are the same as in **A) – C)**.

Scheme S1. Rearrangement pathway for dimeric sandwich POTs based on time-dependent ESI MS (**Figures S13**, **S14**) and UV/vis studies (**Figures S10, S11**). Starting from the tetrasubstituted dimeric sandwich POT **Na{SiW$_{10}$M$_2$}$_2$**, disubstituted monomeric species **{SiW$_{10}$M$_2$}** after **1)** 1 day in H_2O and **2)** 1 day in 100 mM NaOAc/AcOH (pH 5.5). The disubstituted monomeric representatives further hydrolyze to monosubstituted monomers ***α*-{SiW$_{11}$M}** after incubation **3)** in 100 mM NaOAc/AcOH (pH 5.5) for one week, whereas no change is observed in the pristine disubstituted monomers **{SiW$_{10}$M$_2$}** after **4)** one week in H_2O (M = Co^{II}, Ni^{II}). Black, blue and red spheres represent the Si^{IV}, M^{II} and oxygen ions, respectively. Magenta octahedra for {WO$_6$}. The exact pathway for **{SiW$_9$Co$_4$}** cannot be identified by ESI-MS due to the presence of the acetate group in the POT structure; however, the time-dependent UV/Vis spectra (**Figure S10B**) in sodium acetate buffer clearly indicate POT rearrangement.

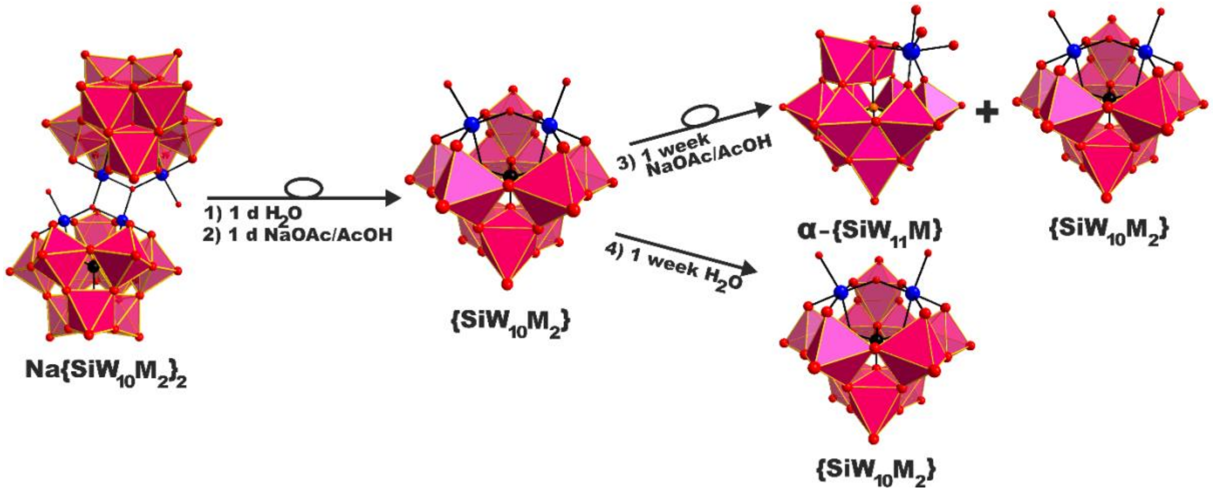

9. POT - Proteinase K co-crystallization

9.1. Preparation of Proteinase K for crystallization

Standard chemicals at least of analytical grade (Sigma Aldrich) were used throughout this study. Proteinase K from *Tritirachium album* was purchased from Sigma-Aldrich (P6556), dissolved in 10 mM Tris/HCl pH 7.0, 0.05 % (m/v) NaN_3 to obtain a 100 g/L protein solution and centrifuged for 15 min at 20817 x g (14000 rpm) to spin down traces of insoluble material. The supernatant was considered sufficiently pure for crystallization trials without further purification (**Figures S15**, **S16**).

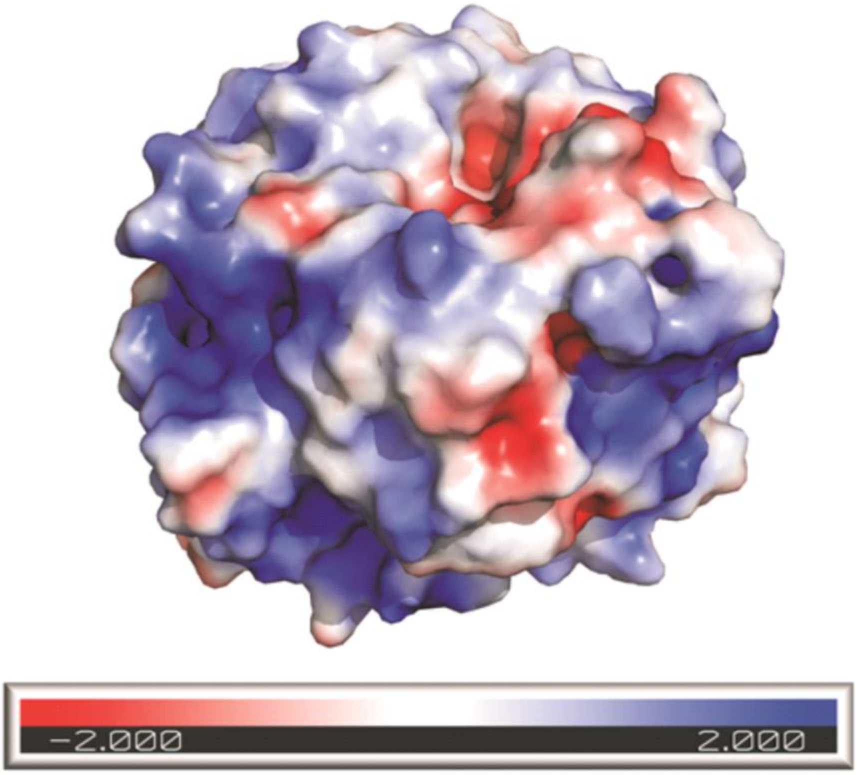

Figure. S15. Electrostatic surface representation of proteinase K. The surface of proteinase K is dominated by positive patches shown in blue. Negative patches are colored in red and patches with no preferred polarity in white. The polarity range is given in the color bar (in units of k_BT/e). Negatively charged POTs are supposed to interact with the protein surface close to the blue positively charged cavities.

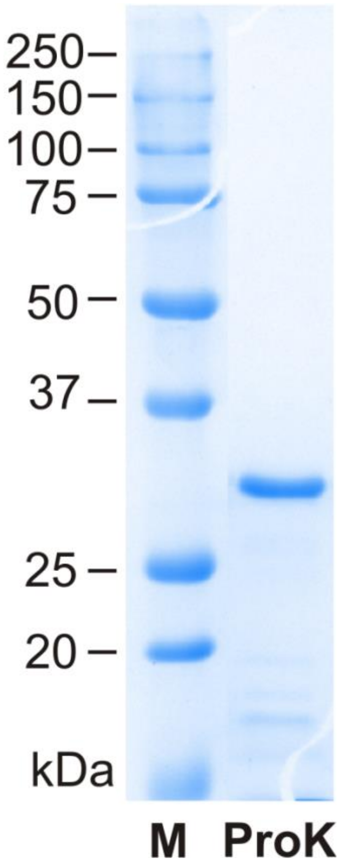

Figure. S16. SDS-PAGE of proteinase K. The protein band is sufficiently pure and migrates at the expected molecular weight of 28.93 kDa. Lane: M: Precision Plus Protein Standard (BIO-RAD).

9.2. Preparation of Polyoxotungstate-Proteinase K co-crystals

Crystals with substituted Keggin POT derivatives were obtained by the hanging-drop vapor diffusion technique in 100 mM NaOAc/AcOH pH 5.5 with 0.7-1.1 M $(NH_4)_2SO_4$ as a precipitant (reservoir solution, 750 µL). For stock solutions, **Na{SiW$_{10}$Co$_2$}$_2$** and **Na{SiW$_{10}$Ni$_2$}$_2$** were dissolved in water, **{SiW$_9$Co$_4$}** was dissolved in 1 M NaOAc/AcOH pH 6.0 to prevent loss of the acetato ligands. Drops were set up from 1 µL of 100 g/L (3.5 mM) proteinase K stock solution, 0.5 µL of 5 mM Keggin POT stock solution and 1 µL of reservoir solution. After few hours, a fine protein precipitate formed due to unspecific aggregation, from which spindle-shaped crystals appeared after 2 weeks. These crystals effected resolubilization of precipitated protein, which then fed crystal growth leading to 50-100 µm crystals. An alternative crystallization condition consisted of 100 mM NaOAc/AcOH pH 5.5 with 0.3-0.7 M $(NH_4)_2SO_4$ and 0.5 M betaine. The zwitterionic additive betaine was successfully applied to prevent the strong unspecific charge-driven aggregation observed for concentrated POT-protein mixtures in the acidic milieu and facilitate the growth of large, ordered crystals, which helped in co-crystallization with **Na{SiW$_{10}$Ni$_2$}$_2$**. Protein crystals were harvested in nylon loops, quickly wiped through a cryo-protectant solution containing 15% (v/v) glycerol and flash-frozen in liquid nitrogen. The crystals were analyzed with a Bruker D8 VENTURE X-ray diffractometer equipped with a multilayer monochromator, a PHOTON II

charge-integrating pixel array detector, a CuK_{α} Incoatec Microfocus (sealed tube) and a Kryoflex cooling device. For data collection and refinement statistics refer to **Table S6**. Note that POT : protein ratios other than 1.4 : 1 either resulted in no crystal formation or precipitates.

9.3. X-ray data collection, structure solution and refinement

After extraction of the measurement parameters by Tim Grüne's sfrmtools (https://homepage.univie.ac.at/tim.gruene/research/programs/conv/sfrmtools/), X-ray data were processed with XDS[24], and the structures were solved by molecular replacement with the PDB protein structure 1ic6 using PHASER[25] within the PHENIX[26] program suite. Structures were refined with phenix.refine (PHENIX) and manually fixed in Coot[27]. After the first refinement steps, it became obvious also by crystal structure analysis that all Keggin derivatives had undergone structural rearrangement to the monosubstituted anions *α*-$[SiW_{11}O_{39}\{Co(H_2O)\}]^{6-}$ (***α*-{SiW$_{11}$Co}**) and *α*-$[SiW_{11}O_{39}\{Ni(H_2O)\}]^{6-}$ (***α*-{SiW$_{11}$Ni}**). Coordinates and restraints files for the *α*-Keggin structure (from PDB entry) were created using phenix.elbow (PHENIX) and the POT ligands were modelled in Coot. Parameters of data collection are given in **Table S6**, together with the refinement statistics. The *B*-factors for the structures with Keggin-POTs ***α*-{SiW$_{11}$Co}** and ***α*-{SiW$_{11}$Ni}** do not significantly differ from the corresponding protein *B*-factors, supporting the proper refinement of X-ray structures and the covalent bond formation between POT and protein.

Table S6. X-ray structural analysis, data processing and refinement. The PDB entry 7A9F contains ***α*-{SiW$_{11}$Co}**, which arose from ***α*-{SiW$_{10}$Co$_2$}$_2$** during the crystallization in the buffer and PDB entry 7A9M contains ***α*-{SiW$_{11}$Co}**, which was formed from ***α*-{SiW$_9$Co$_4$}(OAc)$_3$** during crystallization in the buffer.

Keggin POT	***α*-[SiW$_{11}$Co]$^{6-}$**	***α*-[SiW$_{11}$Ni]$^{6-}$**	***α*-[SiW$_{11}$Co]$^{6-}$**
Crystal data	85 A 1 prok25c1	85 A 4 betain prok27a2	85 A 6 27 prok27b3
**Space group**	$P4_32_12$	$P4_32_12$	$P4_32_12$
***a, b, c,* [Å]**	68.13, 68.13, 102.79	68.01, 68.01, 102.58	68.05, 68.05, 102.47
***α, β, γ* [°]**	90, 90, 90	90, 90, 90	90, 90, 90
**Molecules per asymmetric unit**	1	1	1
**Matthews coefficient [Å^3 Da^{-1}]**	1.90	1.89	1.89
**Solvent content [%]**	0.353	0.349	0.350
**Maximum resolution [Å]**	1.62	1.62	1.62
Data collection and processing			
**Wavelength [Å]**	1.54184	1.54184	1.54184
**Resolution limits [Å]**	41.03 - 1.62 (1.678 - 1.62)	34.01 - 1.62 (1.678 - 1.62)	40.93 - 1.62 (1.678 - 1.62)
**Number of observed reflections**	272438 (5646)	365494 (7386)	364316 (7314)
**Number of unique reflections**	51332 (2798)	56070 (3962)	56364 (4059)
**Redundancy**	5.3 (2.1)	6.5 (1.9)	6.5 (1.8)
***R*$_{p.i.m.}$[a]**	0.0456 (0.5173)	0.03254 (0.4144)	0.0205 (0.1954)

**R_{merge}[b]**	0.09651 (0.6594)	0.08 (0.5361)	0.05134 (0.2387)
**$CC_{1/2}$**	0.994 (0.551)	0.997 (0.704)	0.999 (0.91)
**CC***	0.999 (0.843)	0.999 (0.909)	1 (0.976)
**Completeness [%]**	87.71 (47.83)	96.02 (68.23)	99.79 (99.84)
**$<I/\sigma I>$**	14.19 (1.21)	16.66 (1.33)	23.82 (2.69)
Anomalous signal			
**SigAno [c]**	1.434	1.225	1.519
**AnomCorr [d] [%]**	51	38	55
Refinement statistics			
**Resolution [Å]**	41.03 - 1.62	34.01 - 1.62	40.93 - 1.62
**Reflections used**	51539 (2795)	56064 (3958)	56362 (4058)
**R_{work} [e] [%]**	0.1428 (0.2687)	0.1469 (0.2509)	0.1317 (0.1735)
**R_{free} [f] [%]**	0.1747 (0.3078)	0.1889 (0.2944)	0.1666 (0.2254)
**Number of water molecules**	356	395	376
**Average *B*-factor [$Å^2$]**	11.17	13.06	12.14
***B*-factor : Protein chain**	9.44	9.15	8.75
***B*-factor : ligands (POTs, sulphate)**	13.50	48.90	39.09
***B*-factor : water molecules**	20.32	23.26	22.38
Ramachandran plot[g]			
**Most favoured regions [%]**	97.11	97.47	97.11
**Additional allowed regions [%]**	2.53	2.53	2.53
**Disallowed regions [%]**	0.36	0	0.36
**PDB ID**	7A9F	7A9K	7A9M

[a] $R_{p.i.m.} = \Sigma_{hkl}\{1/[N(hkl) - 1]\}^{1/2} \cdot \Sigma_i |I_i(hkl) - \langle I(hkl)\rangle| / \Sigma_{hkl}\Sigma_i I_i(hkl)$, with $I_i(hkl)$ being the *i*th observation of reflection *hkl*, and $\langle I(hkl)\rangle$ the weighted average intensity for all observations of reflection *hkl*.

[b] $R_{merge} = \Sigma_{hkl}\Sigma_i |Ii(hkl)i - \langle I(hkl)\rangle| / \Sigma_{hkl}\Sigma_i Ii(hkl)_i$.

[c] Mean anomalous difference in units of estimated standard deviation ($|F(+)|-|F(-)|/\sigma$) with the structure factors $F(+)$ and $F(-)$ obtained from the merged intensity observations in each parity class.

[d] Percent correlation between random half-sets of anomalous intensity differences.

[e] $R_{work} = \Sigma|F_{calc}| - |F_{obs}| / \Sigma|F_{obs}|$ x 100, where *F*calc and *F*obs are the calculated and observed structure factor amplitudes, respectively.

[f] R_{free} is calculated using a randomly chosen reference set of 5% of all the reflections collected for each data set.

[g] Calculated with Coot.[27]

9.4. Binding positions of Keggin anions

The two main interaction positions of Keggin-POTs on the surface of proteinase K (**Figue S17**) are involved in various interaction modes with non-polar, positively and negatively polarized surface patches. The two Keggin POTs substituted by Co^{II} and Ni^{II} (monosubstituted **α-{$SiW_{11}Co$}** and **α-{$SiW_{11}Ni$}**) shared a common position on the proteinase K surface, forming a covalent bond to the aspartate side-chain D207 (**position 1**) (**Figure S18A**). The bridging contacts to three different protein molecules are depicted in Fig. S10A. The Keggin anions were also observed in the vicinity of the residue S45 (**position 2**) in a second common site. In this position, the POT clusters interacted with two distinct protein molecules (**Figure S18B**).

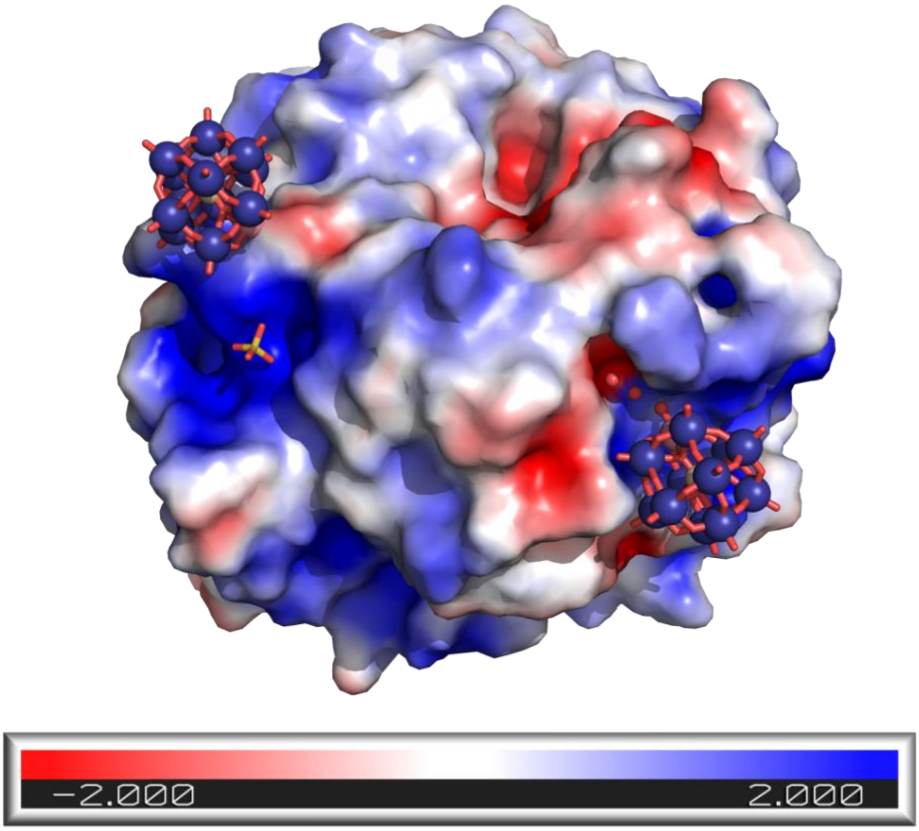

Figure S17. Electrostatic surface representation of proteinase K. This representative protein structure (PDB entry: 7A9F) shows the two main interaction sites of Keggin POTs (here *α*-$[SiW_{11}O_{39}\{Co(H_2O)\}]^{6-}$) on the surface of proteinase K, in the same orientation as presented in **Figure S15**. Positive surface patches are shown in blue, negative patches in red and patches with no preferred polarity in white. The polarity range is shown in the color bar (in units of k_BT/e). The POT, water and sulphate ligands are depicted as ball-and-stick structures with dark blue for W, red for O, ivory for Si, rose for Co and yellow for S. The POT anions turned out to bind to the same surface cavities as previously observed with similar anions, illustrating a certain binding preference of Keggin POTs with moderate charge density to these sites of mixed polarity.

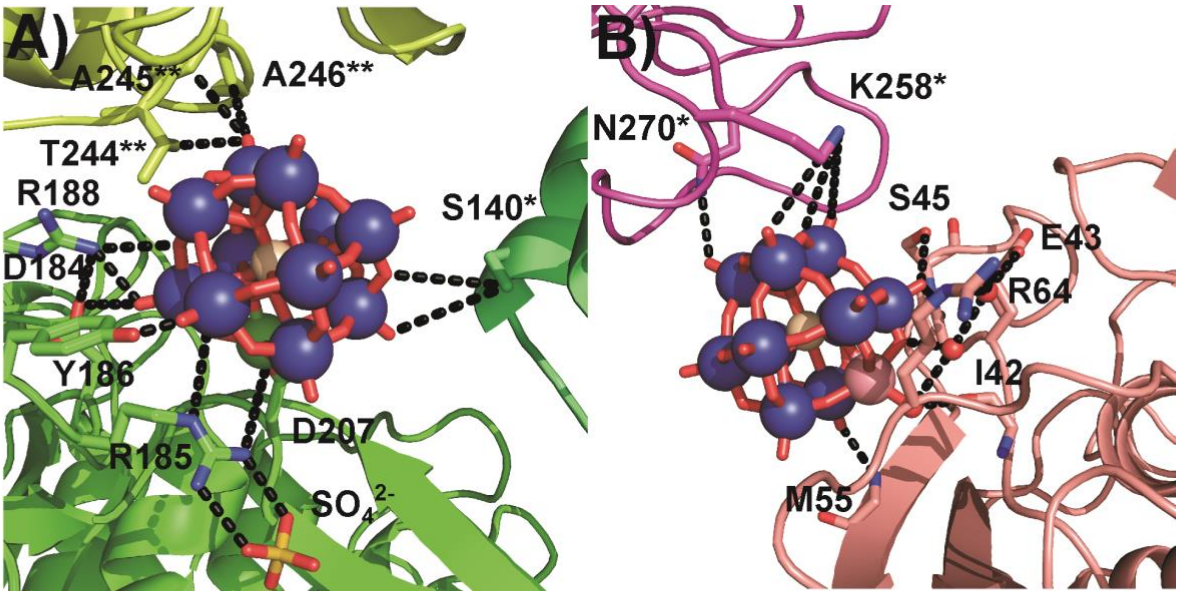

Figure S18. Comprehensive view of Keggin POT interactions with different protein monomers. Distinct protein molecules are represented by different shades of green or red, respectively, and the side chains belonging to the same protein monomer are marked correspondingly with asterisks. One-letter code for amino acids: A, alanine; D, aspartic acid; E, glutamic acid; I, isoleucine; K, lysine; M, methionine; N, asparagine; R, arginine; S, serine; T, threonine; Y, tyrosine. **A)** Protein interactions around the covalent bond to D207 (**position 1**). As a representative structure, ***α*-{SiW$_{11}$Ni}** (PDB entry: 7A9K) was selected. The position of the polyanion is stabilized by main-chain amide and side-chain hydroxyl H-bonding to two more protein chains. **B)** Protein interactions in the vicinity of the H-bonding network close to S45 (**position 2**). As a representative structure, ***α*-{SiW$_{11}$Co}** (PDB entry: 7A9F) was selected. A second proteinase K molecule donates H-bonds to the POT cluster from the opposite side.

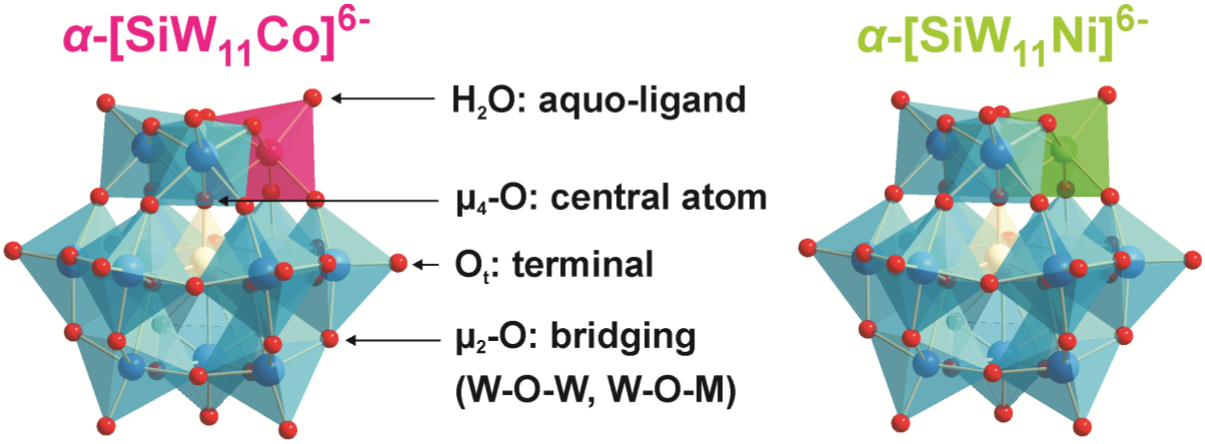

Figure S19. Structures of Keggin POTs as revealed from protein co-crystals and proposed by ESI-MS. The connectivity of the POT architecture is visualized by an overlay of a ball-and-stick and a transparent polyhedron model. The central Si (ivory) tetrahedron is enclosed by twelve tungsten atoms, shown as big blue spheres, and surrounded by light blue octahedra, the corners of which are formed by oxygen atoms (red spheres) of various kinds (bridging or terminal). The substituted transition metals (M) are depicted in colors referring to their octahedral complexes (Co^{II}: red, Ni^{II}: green), and this color-code is conserved in all following

figures. As revealed by the protein-POT co-crystal structures presented here later, the Co- and Ni-containing POTs featured an aquo-ligand in distorted octahedral geometry.

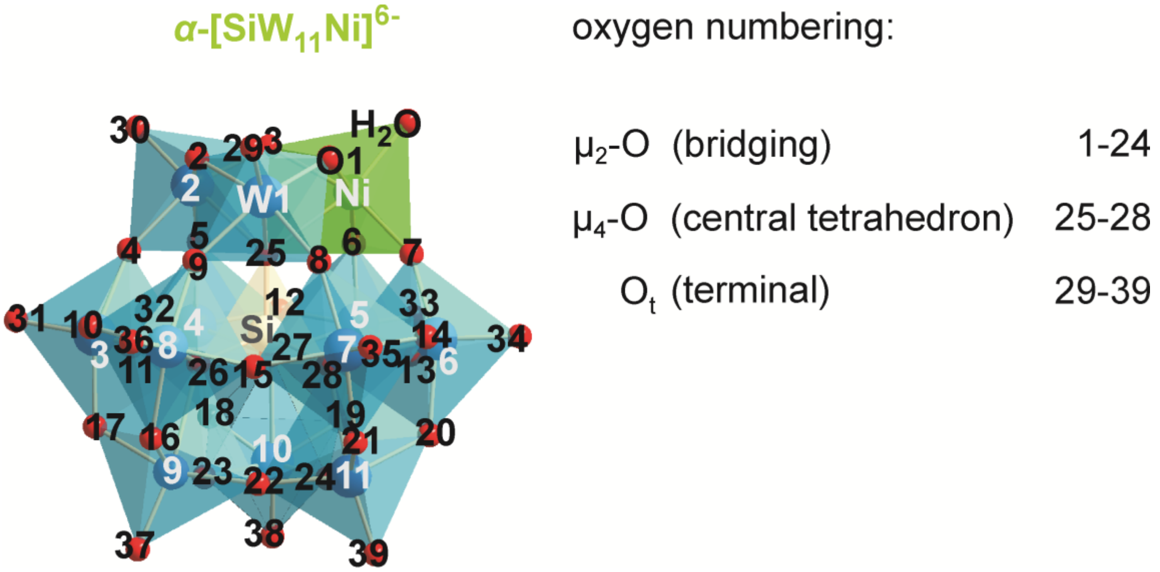

Figure S20. Atom numbering of the representative POT ***α*-{$SiW_{11}Ni$}**. The presented atom numbering provides the basis for the analysis of POT-protein interactions in **Tables S8** and **S9**. Metal atoms are numbered in light grey and oxygen atoms in black.

Table S7. Charge densities and stability in H_2O and NaOAc / HOAc pH 5.5 based on ESI MS and UV/Vis data of Co- and Ni-substituted Keggin silicotungstates.

POT anion	Abbreviation	Charge density *q/m**	Stability in H_2O	Stability in NaOAc / HOAc pH 5.5	Present in****
$[(A\text{-}\alpha\text{-}SiW_{10}O_{37})_2\{Co_4(OH)_2(H_2O)_2\}]^{14-}$	**{$SiW_{10}Co_2$}$_2$**	14 : 24 = 0.58	No	No	Solid sate
$[(A\text{-}\alpha\text{-}SiW_{10}O_{37})_2\{Ni_4(OH)_2(H_2O)_2\}]^{14-}$	**{$SiW_{10}Ni_2$}$_2$**	14 : 24 = 0.58	No	No	Solid sate
$[A\text{-}\alpha\text{-}SiW_9O_{34}(OH)_3\{Co_4(OAc)_3\}]^{8-}$	**{SiW_9Co_4}**	8 : 13 = 0.61	n.d.**	n.d.**	n.d.**
$[SiW_{10}Co_2O_{38}]^{8-}$	**{$SiW_{10}Co_2$}**	8 : 12 = 0.67	Yes	Yes	H_2O and NaOAc / HOAc pH 5.5
$[SiW_{10}Ni_2O_{38}]^{8-}$	**{$SiW_{10}Ni_2$}**	8 : 12 = 0.67	Yes	Yes	H_2O and NaOAc / HOAc pH 5.5
$[SiW_{11}CoO_{39}]^{6-}$	**{$SiW_{11}Co$}**	6 : 12 = 0.5	n.d. ***	Yes	NaOAc / HOAc pH 5.5
$[SiW_{11}NiO_{39}]^{6-}$	**{$SiW_{11}Ni$}**	6 : 12 = 0.5	n.d. ***	Yes	NaOAc / HOAc pH 5.5

* *q* – net charge of POT anion; m – number of metal centers; ** n.d. – no data available due to the unsuitability for ESI-MS; *** n.d. – no data available since monosubstituted anions were not detected in pure aqueous solutions; **** valid for this study.

Table S8. Stabilizing protein interactions in **position 1** (**Figure 2**) for the protein crystal structures with ***α*-{SiW$_{11}$Co}** (PDB entries: 7A9F and 7A9M) and ***α*-{SiW$_{11}$Ni}** (PDB entry: 7A9K), including hydrogen bonds. The POT atoms are numbered according to **Figure S19.**

Ligand	Coordinating POT oxygen	Coordinating protein side chain	Distance [Å]		
			***α*-[SiW$_{11}$Co]$^{6-}$ (7A9F)**	***α*-[SiW$_{11}$Ni]$^{6-}$ (7A9K)**	***α*-[SiW$_{11}$Co]$^{6-}$ (7A9M)**
POT	aquo-ligand replaced	Asp 207 (Oδ2, covalent bond),	1.7 (Oδ2-Co)	1.3 (Oδ2-Ni)	1.8 (Oδ2-Co)
POT	O 3 (μ_2-bridging)	Arg 185 (Nε)	3.9	4.0	4.0
POT	O 32 (terminal)	Tyr 186 (Oη)	1.8	1.8	1.7
POT	O 30 (terminal)	Asp 184 (Oδ2)	3.6	3.6	3.6
POT	O 30 (terminal)	Arg 188 (Nη2)	3.0	2.7	2.6
POT	O 4 (μ_2-bridging)	Arg 188 (Nη2)	3.5	3.4	3.5

Table S9. Stabilizing protein interactions within the hydrogen-bonding network at **position 2** (**Figure 3**) for the protein crystal structures with ***α*-{SiW$_{11}$Co}** (PDB entries: 7A9F and 7A9M) and ***α*-{SiW$_{11}$Ni}** (PDB entry: 7A9K). The POT atoms are numbered according to **Figure S19.**

Ligand	Coordinating POT oxygen	Coordinating protein side chain	Distance [Å]	Distance [Å]	Distance [Å]
			***α*-[SiW$_{11}$Co]$^{6-}$ (7A9F)**	***α*-[SiW$_{11}$Ni]$^{6-}$ (7A9K)**	***α*-[SiW$_{11}$Co]$^{6-}$ (7A9M)**
POT + H_2O 1	aquo-ligand	-	2.1	3.3	2.1
POT	aquo-ligand	Ile 42 (O)	2.7	2.6	2.6
POT	O 33 (terminal)	Met 55 (N)	2.7	2.7	2.7
POT	O 3 (μ_2-bridging)	Ala 44 (N)	3.4	3.4	3.4
POT	O 30 (terminal)	Ser 45 (N)	3.0	3.0	3.0
POT	O 30 (terminal)	Ser 45 (Oγ)	2.7	2.5	2.4
H_2O 1 + H_2O 2	-	-	3.1	3.3	3.3
H_2O 2 + H_2O 3	-	-	2.9	2.8	2.7
H_2O 3	-	Glu 43 (Oε2)	2.6	2.7	2.6
H_2O 3	-	Arg 64 (Nε)	4.0	4.0	4.0
H_2O 3	-	Arg 64 (Nη2)	4.9	5.1	5.1

10. References

1 Tézé, A.; Hervé, G.; Finke, R. G. and D. K. Lyon, in *Inorganic Syntheses*, ed. A. P. Ginsberg, John Wiley & Sons, Inc., Hoboken, **1990**, vol. *27*, pp. 85–96.

2 Bruker SAINT V8.32B Copyright © 2005-2015 Bruker AXS.

3 Sheldrick, G. M. **1996**. *SADABS.* University of Göttingen, Germany.

4 Sheldrick, G. M. **1996**. SHELXS. University of Göttingen, Germany.

5 Sheldrick, G. M. A short history of SHELX. *Acta Cryst.* **2008**, *A64*, 112–122. DOI: 10.1107/S0108767307043930

6 Dolomanov, O. V.; Bourhis, L. J.; Gildea, R. J.; Howard, J. A. K.; Puschmann, H. OLEX2: a complete structure solution, refinement and analysis program. *J. Appl. Cryst.* **2009**, *42*, 339–341. DOI: 10.1107/S0021889808042726

7 Huebschle, C. B.; Sheldrick, G. M.; Dittrich, B. ShelXle: a Qt graphical user interface for SHELXL. *J. Appl. Cryst.* **2011**, *44*, 1281–1284. DOI: 10.1107/S0021889811043202

8 Tocilj, A.; Schlünzen, F.; Janell, D.; Glühmann, M.; Hansen, H. A. S.; Harms, J.; Bashan, A.; Bartels, H.; Agmon, I.; Franceschi, F.; Yonath, A. *Proc. Natl. Acad. Sci.* **1999**, *96*, 14252–14257. DOI: 10.1073/pnas.96.25.14252

9 Rudenko, G.; Henry, L.; Henderson, K.; Ichtchenko, K.; Brown, M. S.; Goldstein, J. L.; Deisenhofer, J. Structure of the LDL Receptor Extracellular Domain at Endosomal pH, *Science* **2002**, 298, 2353–2358. DOI: 10.1126/science.1078124

10 Schemberg, J.; Schneider, K.; Demmer, U.; Warkentin, E.; Müller, A.; Ermler, U. Towards biological supramolecular chemistry: a variety of pocket-templated, individual metal oxide cluster nucleations in the cavity of a Mo/W-storage protein. *Angew. Chem., Int. Ed.* **2007**, *46*, 2408– 2413. DOI: 10.1002/anie.200604858

11 Mauracher, S. G.; Molitor, C.; Al-Oweini, R.; Kortz, U.; Rompel, A. Latent and Active *Ab*PPO4 Mushroom Tyrosinase Cocrystallized with Hexatungstotellurate(VI) in a Single Crystal. *Acta Cryst. D* **2014**, *70*, 2301–2315. DOI: 10.1107/S1399004714013777

12 Mauracher, S. G.; Molitor, C.; Al-Oweini, R.; Kortz, U.; Rompel, Crystallization and preliminary X-ray crystallographic analysis of latent isoform PPO4 mushroom (*Agaricus bisporus*) tyrosinase. *Acta Cryst. F* **2014**, *70*, 263–266. DOI: 10.1107/S2053230X14000582

13 Bijelic, A.; Molitor, C.; Mauracher, S. G.; Al-Oweini, R.; Kortz, U.; Rompel, A. Hen Egg-White Lysozyme Crystallisation: Protein Stacking and Structure Stability Enhanced by a Tellurium(VI)-Centred Polyoxotungstate. *ChemBioChem* **2015**, *16*, 233–241. DOI: 10.1002/cbic.201402597

14 Molitor, C.; Mauracher, S. G.; Rompel, A. Crystallization and Preliminary Crystallographic Analysis of Latent, Active and Recombinantly Expressed Aurone Synthase, a Polyphenol Oxidase, from *Coreopsis grandiflora*. *Acta Cryst. F* **2015**, *71*, 746–751. DOI: 10.1107/S2053230X15007542

15 Molitor, C.; Mauracher, S. G.; Rompel, A. Aurone Synthase Is a Catechol Oxidase with Hydroxylase Activity and Provides Insights into the Mechanism of Plant Polyphenol Oxidases. *Proc. Natl. Acad. Sci.* **2016**, *113*, E1806–E1815. DOI: 10.1073/pnas.1523575113

16 Molitor, C.; Bijelic, A.; Rompel, A. In Situ Formation of the First Proteinogenically Functionalized $[TeW_6O_{24}O_2(Glu)]^{7-}$ Structure Reveals Unprecedented Chemical and Geometrical Features of the Anderson-Type Cluster. *Chem. Commun.* **2016**, *52*, 12286–12289. DOI: 10.1039/C6CC07004C

17 Sap, A.; De Zitter, E.; Van Meervelt, L.; Parac-Vogt, T. N. Structural Characterization of the Complex between Hen Egg-White Lysozyme and Zr^{IV}-

Substituted Keggin Polyoxometalate as Artificial Protease. *Chem*. *Eur. J*. **2015**, *21*, 11692–11695. DOI: 10.1002/chem.201501998

18 Vandebroek, L.; De Zitter, E.; Ly, H. G. T.; Conić, D.; Mihaylov, T.; Sap, A.; Proost, P.; Pierloot, K.; Van Meervelt, L.; Parac-Vogt, T. N. Protein-Assisted Formation and Stabilization of Catalytically Active Polyoxometalate Species. *Chem*. *Eur. J*. **2018**, *24*, 10099–10108. DOI: 10.1002/chem.201802052

19 Breibeck, J.; Bijelic, A.; Rompel, A. Transition Metal-Substituted Keggin Polyoxotungstates Enabling Covalent Attachment to Proteinase K upon Co-Crystallization. *Chem. Commun.* **2019**, *55*, 11519–11522. DOI: 10.1039/C9CC05818D

20 Lisnard, L.; Mialane, P.; Dolbecq, A.; Marrot, J.; Clemente-Juan, J. M.; Coronado, E.; Keita, B.; de Oliveira, P.; Nadjo, L.; Sécheresse, F. Effect of cyanato, azido, carboxylato, and carbonato ligands on the formation of cobalt(II) polyoxometalates: characterization, magnetic, and electrochemical studies of multinuclear cobalt clusters. *Chem. Eur. J.* **2007**, *13*, 3525–3536. DOI: 10.1002/chem.200601252

21 Thouvenot, R.; Fournier, M.; Franck, R.; Rocchiccioli-Deltcheff, C. Vibrational investigations of polyoxometalates. 3. Isomerism in Molybdenum(VI) and Tungsten(VI) compounds related to the Keggin structure. *Inorg. Chem.* **1984**, *23*, 598–605. DOI: 10.1021/ic00144a006

22 Haider, A.; Bassil, B. S.; Lin, Z.; Ma, X.; Haferl, P. J.; Bindra, J. K.; Kinyon, J.; Zhang, G.; Keita, B.; Dalal, N. S.; Kortz, U. Synthesis, structure, electrochemistry and magnetism of cobalt-, nickel- and zinc-containing $[M_4(OH)_3(H_2O)_2(\alpha\text{-}SiW_{10}O_{36.5})_2]^{13-}$ (M = Co^{2+}, Ni^{2+}, and Zn^{2+}). *Dalton Trans*. **2021**, *50*, 3923–3930. DOI: 10.1039/D0DT03392H

23 Antosiewicz, J. M.; Shugar, D. UV–Vis Spectroscopy of Tyrosine Side-Groups in Studies of Protein Structure. Part 2: Selected Applications. *Biophys. Rev.* **2016**, *8*, 163–177. DOI: 10.1007/s12551-016-0197-7

24 Kabsch, W. XDS. *Acta Cryst D.* **2010**, *66*, 125–132. DOI: 10.1107/S0907444909047337

25 McCoy, A. J.; Grosse-Kunstleve, R. W.; Adams, P. D.; Winn, M. D.; Storoni, L. C.; Read, R. J. Phaser crystallographic software. *J. Appl. Cryst.* **2007**, *40*, 658–674. DOI: 10.1107/S0021889807021206

26 Adams, P. D.; Afonine, P. V.; Bunkóczi, G.; Chen, V. B.; Davis, I. W.; Echols, N.; Headd, J. J.; Hung, L.-W.; Kapral, G. J.; Grosse-Kunstleve, R. W.; McCoy, A. J.; Moriarty, N. W.; Oeffner, R.; Read, R. J.; Richardson, D. C.; Richardson, J. S.; Terwilliger, T. C.; Zwart, P. H. PHENIX: A comprehensive python-based system for macromolecular structure solution. *Acta Cryst. D* **2010**, *66*, 213–221. DOI: 10.1107/S0907444909052925

27 Emsley, P.; Cowtan, K. Coot: Model-building tools for molecular graphics. *Acta Cryst. D* **2004**, *60*, 2126–2132. DOI: 10.1107/S0907444904019158
